# Measuring carer quality of life in Duchenne muscular dystrophy: a systematic review of the reliability and validity of self-report instruments using COSMIN

**DOI:** 10.1186/s12955-022-01964-4

**Published:** 2022-04-02

**Authors:** Jill Carlton, Philip A. Powell, Project HERCULES Carer Group

**Affiliations:** 1 School of Health and Related Research (ScHARR), University of Sheffield, Regent Court, 30 Regent Street, Sheffield, S1 4DA UK; 2 Duchenne UK, London, UK

**Keywords:** Carer, Quality of life, Systematic review, Reliability, Validity, Duchenne muscular dystrophy, Psychometrics

## Abstract

**Introduction:**

Duchenne muscular dystrophy is a rare, progressive, life-limiting genetic neuromuscular condition that significantly impacts the quality of life of informal caregivers. Carer quality of life is measured using heterogeneous self-report scales, yet their suitability for Duchenne remains unclear. This review aimed to identify and evaluate the reliability and validity of quality of life instruments in Duchenne carers.

**Materials and methods:**

Systematic searches were conducted in Embase, MEDLINE, CINAHL, PsycINFO, Cochrane Library and Google Scholar. Full research articles reporting data on multiple-item self-report quality of life instruments in informal Duchenne carers were included. Extracted evidence was qualitatively synthesised and evaluated, including risk of bias, against the Consensus-based Standards for the selection of health Measurement Instruments. Duchenne carer collaborators (N = 17) helped rate the instruments’ content validity.

**Results:**

Thirty-one articles featuring thirty-two quality of life instruments were included. Content validity was rated as “inconsistent” based on very low quality evidence. For Duchenne carer collaborators, the best instrument was PedsQL Family Impact Module. Only one instrument had evidence for structural validity (rated “unsatisfactory”) and measurement invariance (rated “satisfactory”). Instruments received “satisfactory” ratings for internal consistency and mixed ratings for construct validity and responsiveness. There was no evidence for reliability, measurement error, or criterion validity.

**Discussion:**

Instruments used to measure Duchenne carer quality of life have limited and often inconsistent supportive psychometric evidence. Further work must investigate instruments’ measurement properties in Duchenne carers and/or the development of new tools. In the interim, we recommend considering the PedsQL Family Impact Module based on Duchenne carer ratings.

**Supplementary Information:**

The online version contains supplementary material available at 10.1186/s12955-022-01964-4.

## Introduction

Duchenne muscular dystrophy (DMD) is a rare x-linked genetic neuromuscular condition with an estimated prevalence of 19.8 per 100,000 live male births [1]. People with DMD experience progressive muscle degeneration and weakness with childhood symptom onset as early as age two. The condition manifests in increasing difficulties with ambulation and motor functioning, with eventual cardiovascular and respiratory problems [2]. Improved treatments and standards of care have increased the life expectancy of people with DMD, with those on ventilator support living for a median 31.8 years [3].

As a pervasive and life-limiting condition, DMD has been observed to impact the health-related quality of life (QoL) of people with the condition in multiple ways. Key areas of impact include independence, relationships and social participation, and psychological wellbeing [4]. As well as the impact on people living with the condition, DMD has a notable influence on the QoL of informal caregivers [4]. Duchenne requires substantial caregiving input, which increases over time as functional ability deteriorates [5]. Caring for someone with DMD involves a vast range of caregiving activities and over time comes to include a host of emotional, social, and physical support, including assistance with day-to-day living (e.g., dressing, eating, cleaning, toileting, transfers and mobility) [6]. As primary carers tend to be family members (i.e., parents), any potential impact on their QoL is heightened as they have to learn to cope with a DMD diagnosis, its progressive and pervasive nature, and the knowledge of what that means for them and their loved ones.

Documented effects on QoL of caring for someone with DMD include problems with sleep, psychological wellbeing, relationships, family resources, physical burden, and impact on the wider family [4]. However, other impacts are likely to exist that have not been well captured in existing data. Carer health-related QoL is typically measured using self-report questionnaires. As well as generic QoL instruments that are used in carers and non-carers alike, specific questionnaires have been developed with an aim to assess carer QoL in particular, including the Care Related Quality of Life (CarerQol) instrument and the Caregiver Strain Index (CSI), which have both been used in DMD research [7]. However, at present, there is little evidence to justify the use of any particular generic and/or specific questionnaire for assessing QoL in DMD carers. Reviews on the burden of caregiving exist [4, 6], but none that critically evaluate the reliability and validity of the self-report instruments that have been used to measure it.

As competing instruments exist to assess carer QoL in DMD, without further data and an evaluation of the evidence on the psychometric properties of these measures, it is difficult to ascertain which instruments are most suitable for use in this context. Given the degree and breadth of the impacts of DMD on daily life for people living with the condition and their informal caregivers, it is not known whether available instruments are sufficiently reliable and valid for assessing carer QoL in DMD. In other progressive conditions, such as neurodegenerative diseases, condition-specific carer questionnaires have been advocated for [8]. Getting the tool right when assessing QoL in DMD carers is important, for understanding the scale of the impact on carers themselves and for accurately ascertaining the benefits of new health technologies.

Health technology assessment (HTA) agencies, such as the National Institute for Health and Care Excellence (NICE), promote the inclusion of “all direct health effects for patients or, when relevant carers” in their Guide to the Methods of Technology Appraisal [9]. This includes carer utility values (or resultant quality-adjusted life years [QALYS]), which are often included in the economic evaluation for health technology appraisals, including recently for Ataluren in treating DMD [10]. For one to have confidence that the evidence on carer QoL is accurate and reliable, it is important that the correct instrument to measure QoL (and thus the generated QALYs) is used, and this judgement should be based on supportive psychometric evidence. Such evidence must be collated in order to appropriately justify the use of a particular questionnaire and/or to indicate where future psychometric and instrument development work is needed. A full assessment of reliability and validity includes internal consistency, reliability, measurement error, content validity, construct validity, criterion validity, and responsiveness, as defined as a result of international expert consensus by the COnsensus-based Standards for the selection of health Measurement INstruments (COSMIN) group [11].

The COSMIN approach represents a structured way of assessing the psychometric evidence of available questionnaires across a number of agreed-upon criteria. Content validity is argued to be the most fundamental psychometric property and refers to the extent that the content of a measure adequately reflects the target construct that is being assessed [11]. It can be meaningfully subdivided into three components: relevance, comprehensiveness, and comprehensibility, which can be understood by asking three questions. First, are the items, response options, and recall period used relevant for the construct, target population, and context of use? Second, is the questionnaire fully comprehensive, or are key aspects of the construct of interest missing? Third, is the content of the questionnaire, including the items, understood by the target population as intended [12]? When assessing content validity, the initial development paper(s) on the instrument and the content of the instrument itself are assessed, as well as any content validity studies undertaken in the population of interest [12].

According to COSMIN, the second most important psychometric property is structural validity [13]. Structural validity describes the extent that scores generated from an instrument appropriately reflect the dimensions of the underlying construct being measured [14]. As QoL is usually theorised and assessed as being multidimensional, questionnaires designed to measure QoL should be dutifully assessed to check that they accurately represent the multidimensional structure of QoL in the target population. Alternatively, if instruments are developed to target a specific dimension of QoL, psychometric tests should be conducted to validate that they are unidimensional when completed by the population of interest. If instead questionnaires are used without accompanying evidence of their structural validity in the target population, interpretation of the data (e.g. through the generation of dimension scores) may not be accurate.

COSMIN has provided internationally-consensual definitions of other important measurement properties, which, when good content and structural validity are documented, all contribute to a measure’s psychometric performance [11]. These include internal consistency, or the degree that items measuring the same thing are interrelated with one another; reliability, describing the proportion of variance due to genuine differences among participants; measurement error, relating to the error in a participant’s response not attributable to genuine changes in the construct being measured; construct validity, which includes structural validity, but more broadly covers the extent to which scores of an instrument are consistent with hypothesised internal relationships, relationships to other measures, and/or differences between groups; criterion validity, or the extent to which scores reflect a “gold standard”; and responsiveness, or the ability of the questionnaire to detect change over time in the construct of interest.

This systematic review has been designed to evaluate the content and psychometric properties of instruments used to measure QoL in informal carers of people with DMD using the COSMIN approach [12, 15]. COSMIN methodology is becoming increasingly used within systematic reviews evaluating the quality of QoL measures in particular health contexts [13, 16–19], including in a recent review of self-report measures used to assess QoL in people with DMD [20], which contributed to the rationale for the development of a new condition-specific QoL measure in this population [21].

For the purposes of this review, we define informal carers as someone providing care to a person with DMD with whom they have a non-professional caregiving relationship, including a parent or guardian, or other family member, friend, neighbour, or other relative or non-kin (where a caregiving relationship is defined) [22]. We exclude children (under 16 years of age) and people who are providing care in a formal or professional capacity, such as personal assistants. We also exclude relations (such as siblings) where a caregiver role is not defined or made explicit. Further, we define QoL as multidimensional, featuring components of physical (e.g., pain/discomfort, mobility, fatigue), psychological (e.g., self-esteem, mood), and social (e.g., relationships with others, participation) wellbeing [23]. We operationalise QoL as inherently subjective and thus do not include assessments of objective function that may affect QoL. In this review, we include instruments with multiple items that measure at least one aspect of QoL in informal carers of people living with DMD. The objectives of this review are to:

1. Identify which questionnaires have been used to assess QoL in informal carers of people with DMD.
2. Evaluate the measurement properties, including the strength and quality of evidence, of questionnaires that have been used to measure QoL in informal carers of people with DMD.
3. Make a recommendation for which questionnaire(s) (if any) are best suited to assess QoL in DMD informal caregivers, based on the current evidence, and identify gaps for future work.

## Methods

The protocol for this review was registered with the International Prospective Register of Systematic Reviews (PROSPERO) (registration no: CRD42020200120) and can be accessed at: https://www.crd.york.ac.uk/prospero/display_record.php?ID=CRD42020200120. The manuscript has been written using the PRISMA 2020 reporting guideline and checklist [24].

### Search Strategy and Information Sources

#### Searches

An information specialist was consulted in developing the appropriate search strategy and was responsible for conducting the main database searches. Search terms in this review included:

(i) Duchenne muscular dystrophy (and derivatives);
(ii) a comprehensive list of carer terms;
(iii) a comprehensive search filter developed by the Patient Reported Outcome (PROM) Group at the University of Oxford to identify questionnaires [25];
(iv) questionnaires known to be used in carers of people with DMD based on an earlier rapid review of the literature [4]; and
(v) a validated search filter by the COSMIN group for identifying studies on measurement properties, as recommended by the COSMIN group [26].

A two-stage search was used, where in the first stage the search terms (i) AND (ii) AND ((iii) OR (iv)) were combined to identify all articles using questionnaires to assess QoL in DMD carers. In the second stage, the names of questionnaires identified in stage one were combined with (i) AND (ii) AND (v) to identify articles reporting on the measurement properties of these instruments for DMD carers. No restrictions on date or language were applied to the search strategy. The two-stage search strategy allowed us to identify which instruments have been used and reported in studies of carers of people with DMD, in the absence of any evidence of reliability and validity for their use. Full copies of the searches are contained in Additional file 1.

#### Electronic databases

The electronic databases searched for the systematic review are outlined in Table 1. All databases were searched from inception.

**Table 1:** Electronic databases for the primary searches

Host	Database	Dates covered	Date searched (Stage 1)	Date searched (Stage 2)
Ovid	Ovid MEDLINE(R) and Epub Ahead of Print, In-Process & Other Non-Indexed Citations, Daily and Versions(R)	1946 to Present	8th July 2020	2nd November 2020
Ovid	Embase	1974 to Present	8th July 2020	2nd November 2020
Wiley	Cochrane Database of Systematic Reviews (Cochrane Library)	CDSR 1996 to Present	8th July 2020	2nd November 2020
Wiley	Cochrane Central Register of Controlled Trials (Cochrane Library)	CENTRAL 1898 to Present	8th July 2020	2nd November 2020
EBSCO	CINAHL	1974 to Present	9th July 2020	2nd November 2020
Ovid	PsycINFO	1806 to Present	8th July 2020	2nd November 2020

#### Additional searches

Following recognised approaches [13, 20], we searched Google Scholar (last searched 5th July 2021) with the names of the instruments identified in the database searches and taken forward for review in order to identify potential development papers for assessing content validity.^1^ The first 100 hits on Google Scholar were screened for inclusion. Where development papers were not found in this manner, manual searching of instrument citations in the included papers was conducted. In addition, citation tracking, by means of screening of references (via Scopus) and Google Scholar citations, was conducted on full text research articles (not development papers) meeting the eligibility criteria at Stage 2 (last searched 5th July 2021), as a supplementary measure to identify any additional studies not captured by the database searching [27].

### Eligibility criteria

The following selection criteria was applied to the search results at Stage 1 (identifying instruments):

- Full text original research article (i.e. not including abstracts, editorials, or reviews);
- Published in English;
- At least 75% of the sample, on which data from an instrument was reported, was formed of informal adult carers of people with DMD;
- Used a self-reported, multi-item questionnaire to assess at least one aspect of QoL; and
- Included a questionnaire that was validated in English, with a free/review copy that was available to access.

Additional selection criteria were applied at Stage 2 (evaluation of measurement properties):

- Reports data on at least one measurement property of the instruments identified and taken forward for review in informal carers of people with DMD.
- Development studies on the instruments identified in Stage 1, to assist with the assessment of content validity, were included in any form (i.e. journal article, book chapter, user manual etc.).

### Selection Process

In order to apply the eligibility criteria for the selection of papers from search results, the following steps were performed by two independent reviewers at all stages^2^:

(I) The titles and abstracts of records identified in the Stage 1 searches were screened against the Stage 1 eligibility criteria (as were any additional records in the Stage 2 searches or through citation tracking). Records were selected for full text review if deemed relevant, potentially relevant, or if doubt existed. All records that were selected for review by either reviewer were then subsequently reviewed at full text.
(II) Full text articles identified in (I) were assessed for eligibility using the Stage 1 eligibility criteria. Any discrepancy was resolved through discussion and reasons for exclusion were documented.
(III) Copies of the instruments identified in the articles in (II) were reviewed to ensure they met the eligibility criteria (i.e. assessed an aspect of QoL). If an English free/review copy of the instrument was not available (or not made available upon request) the questionnaire and corresponding article(s) were excluded from review.
(IV) Full text articles meeting the Stage 1 inclusion criteria AND identified as potentially containing measurement properties using the COSMIN filter were screened using the Stage 2 eligibility criteria, using the title and abstract and full text approach as described above.
(V) In order to identify development papers for the instruments identified for review in (II) and (III) and/or any potential missed articles from the database searches, Google Scholar search results, the results of citation tracking, and manual searching for development papers were screened for inclusion, first by title and abstract and then by full text as described above. Further, the citations of two previous reviews were screened for potentially relevant records not otherwise identified in earlier searches [4, 6].
(VI) A manual review of any articles meeting eligibility criteria at Stage 1 was conducted for potential measurement properties that may have been missed by the COSMIN filter.

### Data extraction and quality appraisal

Data extraction was undertaken independently by two reviewers using a pre-prepared data extraction sheet, with consensus reached through discussion. The data extraction sheet was first piloted (on two development paper articles and two measurement property articles), before being revised for further use. Extraction was informed by tools developed by COSMIN on reporting guidance: https://www.cosmin.nl/tools/guideline-conducting-systematic-review-outcome-measures/. A copy of all data extracted (including which data was sought) is in Additional file 2. Data on interpretability or feasibility of questionnaires (e.g. completion time) was not extracted as it was not typically reported.

COSMIN standards, via the COSMIN risk of bias checklist [28], were used to evaluate the methodological quality of instrument development papers and studies on their measurement properties (ranked on a four-point scale: “very good”, “adequate”, “doubtful” and “inadequate”). The checklist was applied independently by two reviewers, with consensus reached through discussion. Total ratings are determined using the lowest rating for any checklist item for that study (i.e. worst score counts).

### Assessment of content validity

The content validity of each instrument was assessed following published COSMIN guidance [12], which involves evaluating and synthesising evidence from three sources:

(I) The quality of the instrument development;
(II) The quality and results of any additional content validity studies (if available); and
(III) An evaluation of the content of the instrument itself by the review team.

Ratings of relevance, comprehensibility, and comprehensiveness were made for each source of evidence separately and could be satisfactory (+), unsatisfactory (−), or indeterminate (?). Ratings for (I) and (II) were initially made independently by two reviewers, and, in the case of disagreement, consensus was reached following discussion.

In order to evaluate the instrument content (III), informal carers (parents) of people with DMD aged between 3 and 19 (identified through Duchenne UK) were included as part of the review team (15 mothers, 2 fathers). The carers rated a selection of instruments on 8 criteria across relevance (5 criteria), comprehensibility (2 criteria), and comprehensiveness (1 criterion). As a significant number of instruments were included in the review, they were distributed across the carer group, so that each instrument was rated by a minimum of three carers. Further, carers provided ratings for full instruments (i.e. how they are usually disseminated), rather than instrument subscales separately. While the COSMIN terminology was retained in rating sheets (for standardisation), elaborated instructions were provided to explain the concepts and ratings in lay terms (see Additional file 3). All documents were handled electronically.

For each criterion carers could provide a rating of “positive” (+), “negative” (−), or “unsure” (?). For example, for comprehensiveness the criterion is “Are all key concepts included?”, rated as yes (+), no (−), or unsure (?). Ratings across reviewers were then synthesised using rules adapted from COSMIN, to account for more than three reviewers, as outlined in Table 2. As we wanted to put greater descriptive emphasis on the results of the review by Duchenne carers, we included two additional possible synthesised ratings: inconsistent trending towards positive (± (+)) and inconsistent trending towards negative (± (−)). These are not traditionally used in COSMIN, so we have included these to provide additional descriptive information for the carer ratings only. Reviewer ratings were then synthesised for each aspect of content validity (i.e. relevance, comprehensiveness, and comprehensibility) using rules defined by COSMIN [12].^3^

**Table 2:** Data synthesis rules for carer reviewer ratings

Criteria	Synthesised rating
≥ 75% of ratings are+/−/?	+/−/?
< 75% of ratings are+/−/?, equal number of+/−	±
< 75% of ratings are+/−/?, greater number of + than −	± (+)
< 75% of ratings are+/−/?, greater number of − than +	± (−)
Only one carer rating is ?	Ignore ? and rate as above
Two carer ratings are ? and only one other carer rating is ±	?

### Assessment of Psychometric Properties

Each source of evidence on the remaining measurement properties was evaluated against the COSMIN criteria for good measurement properties, using the same satisfactory (+), unsatisfactory (−), and indeterminate (?) as mentioned above [15]. The criteria for good measurement properties specifies thresholds for evaluating effect measure(s) for each measurement property and what data was eligible, such as Cronbach’s alpha for internal consistency, with the full list of effect measure(s) evaluated described in the COSMIN manual [15]. All ratings were initially made independently by two reviewers and then ratified, with any disagreement resolved through discussion.

COSMIN ratings for construct validity (convergent and known groups) and responsiveness require a priori criteria for the testing of hypotheses by the review team [15]. These are based on generic hypotheses provided in the COSMIN manual. The hypotheses were based on expected effect size magnitude (of *r* for convergent validity and *d* for between-group tests), which were either reported in the studies or calculated by the reviewers.^4^ COSMIN criteria is for the review team to judge whether ≥ 75% of the results are in accordance with these hypotheses (a + rating). The hypotheses used are in Table 3.

**Table 3:** Generic hypotheses used for the assessment of construct validity and responsiveness

A priori rules for hypothesis testing	
Convergent validity	
1	If construct being measured is the same or similar to that measured by the instrument then the correlation should be *r* ≥ 0.5
2	If construct being measured is dissimilar but related to that measured by the instrument then the correlation should be between *r* = 0.3 and 0.49
3	If construct being measured is judged as unrelated to that measured by the instrument then the correlation should be *r* < 0.3
Known groups validity/responsiveness	
1	Differences between groups where a large difference is expected should be *d* ≥ 0.8
2	Differences between groups where a medium difference is expected should be between *d* = 0.5 and 0.79
3	Differences between groups where a small difference is expected should be between *d* = 0.2 and 0.49
4	Differences where no or a trivial difference is expected should be *d* < 0.2

Reviewers then made a judgment on the size of difference that they would expect given the comparison being made (see Additional file 2). For example, if the Hospital Anxiety and Depression Scale (HADS) depression subscale was compared to another depression measure then a correlation coefficient of *r* ≥ 0.5 was expected. Likewise, if a study compared QoL results for mothers of people with DMD against a control comparison group of mothers (who were not health-related carers), medium or large differences in the expected direction were rated as acceptable (i.e. *d* ≥ 0.5).

### Evidence synthesis

Individual ratings for each measurement property were qualitatively synthesised using a priori rules based on those recommended by COSMIN (see Table 4) [12, 15]. Based on these rules, each instrument could receive an overall (synthesised) rating of sufficient (+), insufficient (−), or inconsistent (±) for each measurement property (with content validity additionally split into relevance, comprehensibility, and comprehensiveness). For example, if the rating of the instrument development was satisfactory (+) and the carer rating was satisfactory (+) for relevance, then the overall synthesised rating for relevance for that questionnaire would be satisfactory (+). Content validity was evaluated for the total instrument (except for the State-Trait Anxiety Inventory [STAI] form X state and trait versions, which were considered separable). Where a total score was not available, subscales of instruments were rated for other measurement properties (where evidence was available).

**Table 4:** Data synthesis rules for each measurement property

Criteria	Synthesised rating
All ratings are+/−/±/?	+/−/±/?
At least one rating is + and one rating is −	±
^a^Development paper is ±, reviewer rating is+/−	+/−
Only one rating is ?	Ignore ? and rate as above
Two or more ratings are ?	?
All other situations	±

^a^Content validity synthesis only

As recommended by COSMIN, a different weight was applied to development studies than the reviewer ratings for the rating of content validity (and its subcomponents) only [12], whereby more weight was chosen to be applied to reviewer ratings than the development study. This contradicts traditional COSMIN recommendations to place more weight on published literature, but was decided upon as carers represent the target population for this review and are likely to a better judge of instruments’ content validity (as the vast majority of instruments were not developed in a Duchenne carer setting).

In the final step, the quality of evidence was evaluated via a modified Grading of Recommendations, Assessment, Development and Evaluations (GRADE) approach [29], and categorised as “high”, “moderate”, “low”, or “very low”. The quality of evidence rating incorporates down-grading based on the risk of bias evaluation noted above (which includes limited or missing evidence); imprecision (based on pooled sample size); inconsistency in evidence; and indirectness (of sources of evidence) [15]. Full details on how all the above criteria are applied are detailed elsewhere in comprehensive COSMIN manuals [12, 15].

## Results

### Searches and study inclusion

**Fig. 1:**
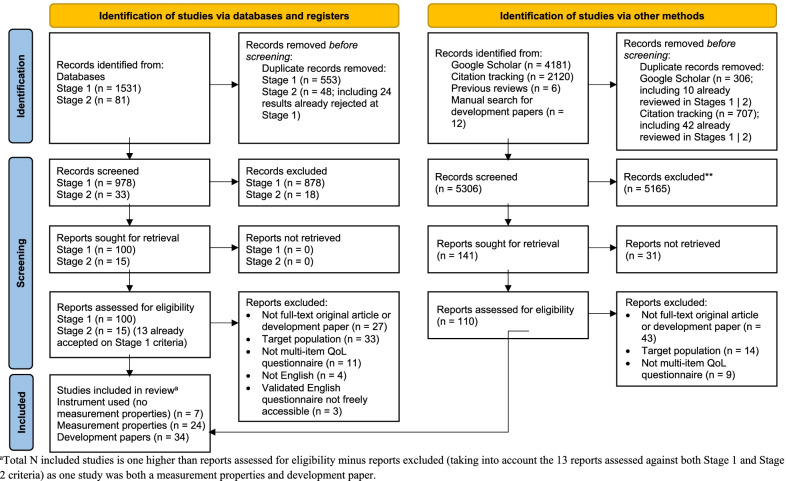
PRISMA flow diagram of study searches (adapted from [24])

The results of the searches and study selection are summarised in the PRISMA diagram in Fig. 1. In Stage 1, a total of 1531 records were identified via database searching, from which 553 duplicates were removed and 978 were screened at title and abstract. A total of 100 were further assessed for eligibility at full text, from which 76 were rejected and 24 were included in the review. Cohen’s kappa of inter-rater reliability for full text review at Stage 1 was *κ* = 0.73, which can be interpreted as ‘substantial’ agreement [30]. In Stage 2, 81 records were identified, from which 48 duplicates were removed and 33 were screened at title and abstract. Fifteen records were assessed for eligibility against the Stage 2 eligibility criteria at full text (13 of which had already been accepted in Stage 1), from which 3 were rejected and 12 were accepted as having evidence of measurement properties. Cohen’s kappa at Stage 2 full text review was *κ* = 0.59 (‘moderate’ agreement). Finally, after removing duplicates, 5306 records were screened from additional sources (i.e., Google Scholar searches, citation tracking, previous reviews, and manual searching for development papers). From these 141 were sought for retrieval and 110 were reviewed at full text (of the 31 not retrieved, 12 were duplicates, 9 were no longer available, 6 were not in English, and 4 were not either a full text article in DMD carers or a development paper). Of the 110 reviewed, 70 were rejected and 40 accepted (7 of these were DMD studies and 33 development papers). Cohen’s kappa for the full text review from additional sources was *κ* = 0.74 (‘substantial agreement’).

In addition to the 15 articles reviewed for measurement properties identified using the COSMIN filter in the Stage 2 database searches, the DMD carer studies added from additional sources or otherwise meeting the eligibility criteria at Stage 1 were manually screened for evidence of measurement properties. This resulted in an additional 12 articles being included in the review with data on at least one measurement property.

To summarise, 31 records were included in the review where a multi-item QoL instrument meeting the inclusion criteria had been used in a published study with DMD carers, 24 of these contained evidence of measurement properties (7 did not). A further 34 development papers of these instruments were included (33 of these came from additional searches and 1 was both a development paper and DMD carer study identified in the primary database searching).

### Questionnaires identified for review

From the searches in Stage 1, 58 questionnaires (from 34 articles) were considered for potential inclusion. After a review of their content, 30 were taken forward for COSMIN review (10 were excluded due to being inaccessible or behind a paywall; 9 were judged as not assessing QoL; 4 were not a caregiver measure; 2 had no validated English version available; 1 was not self-report; 1 was a single-item instrument; and 1 was a duplicate). Two additional instruments were added from the additional sources, giving a total of 32 questionnaires for review. The questionnaires taken forward for review are summarised in Table 5 (see Additional file 4 for a full list of the 60 questionnaires identified in the searches, with reasons for exclusion).

**Table 5:** Summary of the 32 instruments used to assess carer QoL in DMD from the full-texts meeting the Stage 1 eligibility criteria (n = 31)

Instrument	Recall period	N subscales (items)	Total score (Y/N)	Dimensions of QoL assessed (sub-domains)	Response option type (N options)	Origin language (country)	Target population	Intended context of use
12-Item Short Form Survey (SF-12) [31]	Varies by item	2 or 8 (12)	N	Physical health (Physical functioning, role-physical, bodily pain, general health), Mental health (vitality, social functioning, role-emotional, mental health)	Varies by item (varies by item)	English (US)	Adults (general population)	Research
36-Item Short Form Survey (SF-36) [32, 33]^a^	Varies by item	2 or 8 (36)	N	Physical health (Physical functioning, bodily pain, role limitations due to physical health problems, general health), Mental health (role limitations due to personal or emotional problems, emotional well-being, social functioning, energy/fatigue)	Varies by item (varies by item)	English (US)	Adult patients and general population	Research/Clinical
Beck Depression Inventory (BDI) [34]^a^	General/unspecified	0 (21)	Y	Depression	Varies by item (4)	English (US)	Adult patients	Research/Screening
Caregiver Strain Index (CSI) [7]	General/unspecified	0 (13)	Y	Caregiver strain	Agreement (2)	English (US)	Adult carers	Research/Screening
Caregiver Strain Index Plus (CSI +) [7]	1 week	0 (18)	Y	Caregiver strain (including positive aspects of care)	Agreement (2)	Dutch (Netherlands)	Assumed same as CSI	Assumed same as CSI
Caregiver Well-being Scale (CWBS) [35]	3 months	2 (45)	Y^b^	Caregiver wellbeing (Basic human needs, activities of daily living)	Frequency (5)	English (US)	Adult carers	Social work/Practice
Care-related Quality of Life Instrument (CarerQoL) [7]	Present	7 (7 + VAS)	Y	Caregiver burden (fulfillment, relational problems, mental health problems, problems with daily activities, financial problems, support, physical problems)	Severity (4)	Dutch (Netherlands) (assumed)	Informal adult caregivers	Economic evaluation
DUKE Health Profile (DUKE) [36–40]	Varies by item	11 (17)	Y	Health status (Physical health, mental health, social health, general health, perceived health, self-esteem, anxiety, depression, anxiety-depression (DUKE-AD), pain, disability)	Varies by item (3)	English (US)	Adult patients	Research/Clinical
EQ-5D-3L [7, 31, 41–43]^a^	Today	5 (5 + VAS)	Y	Health status (mobility, self-care, usual activities, pain/discomfort, and anxiety/depression)	Severity (3)	Multiple	Adults (general population)	Research
EQ-5D-5L [41]	Today	5 (5 + VAS)	Y	Health status (mobility, self-care, usual activities, pain/discomfort, and anxiety/depression)	Severity (5)	English (UK) and Spanish	Assumed same as 3L	Assumed same as 3L
ENRICHD Social Support Instrument (ESSI) [44]	Present	0 (6 + 1)	Y	Social Support	Frequency (5)	English (US)	Adult patients	Screening
Epworth Sleepiness Scale (ESS) [45]	Recent times	0 (8)	Y	Daytime sleepiness	Probability (4)	English (Australia)	Adult patients	Unclear
Family APGAR (FAPGAR) [34, 36–40]	General/unspecified	0 (5)	Y	Family member's satisfaction	Frequency (3)	English (US)	Adult patients	Screening
Family Problems Questionnaire (FPQ) [46, 47]	2 months	5 (34 + 2)	N	Objective burden, subjective burden, support received by professionals and from members of social network, relative's positive attitude toward the patient, relative criticism of the patient's behaviour (+ indirect costs, burden on children)	Varies by item (4)	Italian (Italy)	Adult carers	Clinical/Screening
Female Sexual Function Index (FSFI) [48]	4 weeks	6 (19)	Y	Female sexual functioning (desire, arousal, lubrication, orgasm, satisfaction, pain)	Varies by item (varies by item)	English (US)	Adult women	Research
Hospital Anxiety and Depression Scale (HADS) [7, 32]^a^	Last week	2 (14)	N	Anxiety, depression	Frequency (4)	English (UK)	Adult patients	Clinical/Screening
Kessler Psychological Distress Scale (K6) [44]	30 days	0 (11)	Y	Non-specific psychological distress	Varies by item (varies by item)	English (US)	Adults (general population)	Research/Screening
PedsQL Family Impact Module (PedsQL FIM) [49, 50]	1 month or 1 week	8 (36)	Y	Family impact (physical functioning, emotional functioning, social functioning, cognitive functioning, communication, worry, daily activities, family relationships)	Frequency (5)	English (US)	Parents	Unclear
Perceived Personal Control Questionnaire (PPC) [51]	General/unspecified	0 (5)	Y	Control	Amount (11)	English (US)	Parents	Unclear
Perceived Stress Scale (PSS) [32]	1 month	0 (10)	Y	Perceived stress	Frequency (5)	English (US)	Adults (general population)	Research
Pittsburg Sleep Quality Index (PSQI) [45, 48, 52]	1 month	7 (19)	Y	Sleep quality (subjective sleep quality, sleep latency, sleep duration, habitual sleep efficiency, sleep disturbances, use of sleeping medications, daytime dysfunction)	Varies by item (varies by item)	English (US)	Adult patients	Research/Clinical
Psychological Adaptation Scale (PAS) [51]	General/unspecified	4 (20)	Y	Adaptation (coping efficacy, self-esteem, social integration, spiritual well-being)	Severity (5)	English (US)	Adult patients and carers	Research/Clinical
Questionnaire on Resources and Stress (QRS) [53, 54]	General/unspecified	15 (285)	Y^b^	Burden (Poor health/mood, excess time demands, negative attitude toward index case, overprotection/dependency, lack of social support, overcommitment/martyrdom, pessimism, lack of family integration, limits on family opportunity, financial problems, physical incapacitation, lack of activities for index case, occupational limitations for index case, social obtrusiveness, difficult personality characteristics)	Agreement (2)	English (US)	Adult carers	Research/Clinical
Satisfaction with Life Scale (SWLS) [54]^a^	General/unspecified	0 (5)	Y	Life satisfaction	Agreement (3)	English (UK)	Unclear	Unclear
Social Networks Questionnaire (SNQ) [46, 47]	Varies by item	4 (15)	N	Quality/frequency of social contacts, practical social support, emotional support, presence/quality of an intimate supportive relationship	Varies by item (varies by item)	Multiple language versions	Adult carers	Unclear
State-Trait Anxiety Inventory (form X) (STAI-X) [55]	Present (state); General (trait)	2 (40)	N	State anxiety, Trait anxiety	Severity (state); Frequency (trait) (4)	English (US)	Adults (general population)	Research/Clinical
Symptom Checklist 90-Revised (SCL-90-R) [56]	1 week	9 or 3 global indices (90)	Y	Global severity (somatization, obsessive–compulsive, interpersonal sensitivity, depression, anxiety, hostility, phobic anxiety, paranoid ideation, psychoticism)	Severity (5)	English (US)	Adult and adolescent patients	Research/Clinical
WHO Quality of Life-BREF (WHOQOL-BREF) [34, 57, 58]^a^	2 weeks	4 (26)	Y^b^	Quality of life (Physical health, psychological, social relationships, environment)	Varies by item (4)	Multiple	Adult patients, carers, and general population	Research/Clinical
Worry about Care for Child with DBMD (WAC-DBMD) [51]	Varies by item	0 (3)	Y	Worry	Severity (5)	English (US)	Parents	Unclear
Zarit Burden Inventory (ZBI) 4-item [44]	General/unspecified	0 (4)	Y	Caregiver burden	Frequency (5)	English (US)	Adult carers	Research/Screening
Zarit Burden Inventory (ZBI) 12 item [51]	General/unspecified	0 (12)	Y	Caregiver burden	Frequency (5)	English (US)	Adult carers	Research
Zarit Burden Inventory (ZBI) 22 item [31, 41, 42, 58–60]	General/unspecified	0 (22)	Y	Caregiver burden	Frequency (5)	English (US)	Adult carers	Unclear

Citations included next to instrument name are the studies included in the review where that questionnaire has been used in DMD carers

^a^Information already extracted and adapted from Powell et al. [20]

^b^Total score not specified by developers, but total score used in an identified DMD carer study

### COSMIN Evaluation of measurement properties

The overall results of the COSMIN evaluation of measurement properties for the instruments included in the review are summarised in Table 6. Full rating sheets on which this evaluation is based are included in Additional file 5. Of note is the lack of published evidence for many measurement properties for these instruments in Duchenne carers across the board. The Zarit Burden Inventory (ZBI) 22-item had the best breadth of evidence, due to a dedicated study exploring selected psychometric properties in carers of people with DMD [60]. However, evidence on responsiveness was still missing. Furthermore, no evidence on reliability, measurement error, or criterion validity was recorded for any of the questionnaires (not shown on Table 6).

**Table 6:** Overall rating and quality of evidence of measurement properties for included instruments against COSMIN criteria

	Content validity		Structural validity		Internal consistency		Hypotheses testing for construct validity		Cross-cultural validity/measurement invariance		Responsiveness	
	COSMIN rating	Quality of evidence	COSMIN rating	Quality of evidence	COSMIN rating	Quality of evidence	COSMIN rating	Quality of evidence	COSMIN rating	Quality of evidence	COSMIN rating	Quality of evidence
BDI^a^	±	Very low	?	None	?	None	?	None	?	None	?	None
Carer QoL	±	Very low	?	None	?	None	?	None	?	None	?	None
CSI	±	Very low	?	None	?	None	−	Very low	?	None	?	None
CSI+	±	Very low	?	None	?	None	?	None	?	None	?	None
CWBS	±	Very low	?	None	+	Moderate	?	None	?	None	?	None
DUKE	±	Very low	?	None	+	High	±	High	?	None	?	None
EQ-5D-3L^a^	±	Very low	?	None	?	None	−	Low	?	None	?	None
EQ-5D-5L	±	Low	?	None	?	None	?	None	?	None	?	None
ESS	±	Very low	?	None	?	None	−	Low	?	None	?	None
ESSI	±	Very low	?	None	?	None	?	None	?	None	?	None
FAPGAR	±	Very low	?	None	+	High	+	High	?	None	?	None
FPQ^b^	±	Very low	?	None	?	High	?	None	?	None	?	None
FSFI	±	Very low	?	None	?	None	?	None	?	None	?	None
HADS^a^	±	Very low	No total score									
HADS (Anxiety)	See HADS for rating of overall measure		?	None	+	Moderate	+	High	?	None	?	None
HADS (Depression)			?	None	+	Moderate	+	High	?	None	?	None
K6	±	Very low	?	None	?	None	?	None	?	None	?	None
PAS	±	Very low	?	None	+	High	−	Very low	?	None	+	Moderate
PedsQL FIM	±	Very low	?	None	?	None	−	Very low	?	None	?	None
PPC	±	Very low	?	None	+	High	?	None	?	None	−	Moderate
PSQI	±	Very low	?	None	?	None	±	Moderate	?	None	?	None
PSS	±	Very low	?	None	+	Moderate	+	Low	?	None	?	None
QRS	±	Very low	?	None	+	Low	+	Moderate	?	None	?	None
SCL-90-R	±	Very low	?	None	?	None	+	Very low	?	None	?	None
SF-12	±	Very low	No total score									
SF-12 (MCS)	See SF−12 for rating of overall measure		?	None	?	None	−	Low	?	None	?	None
SF-36^a^	±	Very low	No total score									
SF-36 (BP/Pain)	See SF−36 for rating of overall measure		?	None	+	Moderate	±	High	?	None	?	None
SF-36 (E|F/VT)			?	None	+	Moderate	±	High	?	None	?	None
SF-36 (EW/MH)			?	None	+	Moderate	+	High	?	None	?	None
SF-36 (GH)			?	None	+	Moderate	+	High	?	None	?	None
SF-36 (MCS)			?	None	?	None	−	Low	?	None	?	None
SF-36 (PCS)			?	None	?	None	+	Low	?	None	?	None
SF-36 (PF)			?	None	+	Moderate	+	High	?	None	?	None
SF-36 (RE)			?	None	+	Moderate	+	High	?	None	?	None
SF-36 (RP)			?	None	+	Moderate	+	High	?	None	?	None
SF-36 (SF)			?	None	+	Moderate	±	High	?	None	?	None
SNQ	±	Very low	No total score									
SNQ (Subscale A)	See SNQ for rating of overall measure		?	None	−	High	−	Very low	?	None	?	None
STAI-X (state)	±	Very low	?	None	?	None	−	Very low	?	None	?	None
STAI-X (trait)	±	Very low	?	None	?	None	−	Very low	?	None	?	None
SWLS^a^	±	Very low	?	None	?	None	+	Very low	?	None	?	None
WHOQOL-BREF^a^	±	Very low	?	None	?	None	±	Moderate	?	None	?	None
WAC-DBMD	±	Very low	?	None	+	High	?	None	?	None	+	Moderate
ZBI (4 item)	±	Very low	?	None	?	None	?	None	?	None	?	None
ZBI (12 item)	±	Very low	?	None	+	High	?	None	?	None	−	Moderate
ZBI (22 item)	±	Very low	−	High	+	High	±	Low	+	Very low	?	None

+, satisfactory results; −, unsatisfactory results; ±, inconsistent results; ?, indeterminate results. No evidence recorded across all instruments for reliability, measurement error, criterion validity, so omitted from this table. BP/Pain, bodily pain/pain subscale; E|F/VT, energy|fatigue/vitality subscale; EW/MH, emotional wellbeing/mental health subscale; GH, general health subscale; MCS, mental component summary score; PCS, physical component summary score; PF, physical functioning subscale; RE, role emotional subscale; RP, Role physical subscale; SF, social functioning subscale

^a^Evidence and rating on the development study extracted from a prior review [20], as per COSMIN guidance [12]

^b^FPQ has no total score, but internal consistency rating was the same for all subscales, as Cronbach’s alpha was presented as a range in the paper

#### Content validity

34 development papers were evaluated using COSMIN methodology, with the development paper ratings from six instruments (Beck Depression Inventory [BDI], EQ-5D-3L, Hospital Anxiety and Depression Scale [HADS], 36-Item Short Form Survey [SF-36], Satisfaction with Life Scale [SWLS], WHO Quality of Life-BREF [WHOQOL-BREF]) extracted from a prior review [20]. Key details from these papers are summarised in Table 7, including the COSMIN rating and whether carers were involved in the development of the instrument. All but two of the instruments (EQ-5D-5L and WHOQOL-BREF) received an inadequate rating for the methodological quality of the development phase. This inadequate rating was primarily driven by the instrument development study not being performed in a sample representing the measure’s target population. In fact, of the instruments in this review only three featured a concept elicitation/development study of some form (Caregiver Strain Index [CSI], EQ-5D-5L, Questionnaire on Resources and Stress [QRS]), content for the rest was derived from reviewing the literature, existing measures, and/or expert/researcher judgment. Strikingly, only two instruments had carers involved in some form in the development of the measure (CSI, WHOQOL-BREF).

**Table 7:** Summary and assessment of development papers for the instruments included in the review

Instrument	Construct definition	Theory, model, conceptual framework or rationale for construct	Development study	
			COSMIN quality rating	Were carers involved? (Y/N)
BDI [62]^a^	“the items were chosen on the basis of their relationship to the overt behavioral manifestations of depression and do not reflect any theory regarding the etiology or the underlying psychological processes in depression”	Unclear	Inadequate	N
CarerQoL [63]	"Subjective [caregiver] burden is a measure of how straining the caregiver experiences the care giving task to be."	"Existing burden measures were studied in order to determine which dimensions could be used to describe the situation surrounding the burden experienced by the caregiver (…) We set out to develop an instrument that was capable of describing the 'care profile' as well as valuing the impact on the caregiver's overall quality of life."	Inadequate	N
CSI [64, 65]	"By strain we mean those enduring problems that have the potential for arousing threat, a meaning that establishes strain and stressor as interchangeable concepts."	Unclear	Inadequate	Y
CSI+ [66]	Assumed same as CSI and in addition: "Positive aspects of care (PACs) are an umbrella term referring to a variety of feelings about, and reactions to, caring. PACs have been described in a number of different ways: gain, satisfaction, rewards, pleasures, positive appraisal, enjoyment, growth, meaning and uplifts."	Unclear	Inadequate	N
CWBS [67]	"Caregivers' satisfaction with basic human needs and activities of daily living"	"The health-strength model described by Weick (1986) and Weick and Freeman (1983) (…) The development of the well-being scale for caregivers began by examining the Weick and Freeman (1983) health menu (…) The questionnaire was also based on Maslow's hierarchy of needs (1968) and scales developed by Barusch (1988), McCubbin (1982), Slivinske and Fitch 1987 and George and Gwyther (1986)"	Inadequate	N
DUKE [68–71]	"Functional health status"	"The principal measures developed for the DUKE are based upon the three WHO dimensions: physical, mental, and social health. Items were selected from the DUHP first to fit these constructs, and then rearranged to form the other measures"	Inadequate	N
EQ-5D-3L [72, 73]^a^	“Health-related quality of life”	“Generic measure should aim to capture physical, mental, and social functioning [6] (…) People have to weigh up the very diverse attributes of health to determine which, on balance, seems best, it should be possible to elicit such overall valuations by some suitable investigatory method which generates a single index value for each health state”	Inadequate	N
EQ-5D-5L [61]	"Health status"	Assumed same as 3L	Doubtful	Unclear
ESS [74]	"Sleep propensity"	"The concept of the ESS was derived from observations about the nature and occurrence of daytime sleep and sleepiness."	Inadequate	N
ESSI [75, 76]	"Social support"	Unclear	Inadequate	N
FAPGAR [77]	"A patient's view of the functional state of his or her family"	"In order to establish the parameters by which a family’s functional health can be measured, five basic components of family function were chosen. These components, which are defined in Table 1. were elected by the author since they appear to represent common themes in the social science literature that deals with families."	Inadequate	N
FPQ [78, 79]	"Family burden and relatives' attitudes"	"The relevant theory is now commonly known as Expressed Emotion (EE) (Brown et al., 1972; Vaughn and Leff, 1976a; Leff and Vaughn, 1985) (…) Most of these studies followed the Hoenig & Hamilton (1966, 1969) distinction between «objective» burden (practical problems and difficulties, e.g. effects on relatives' finances, on work, on social life) and «subjective» burden (the subjective distress which relatives attribute to the presence of the patient in their life). "	Inadequate	N
FSFI [80]	"Female sexual arousal and other relevant domains of sexual functioning in women"	"Recently, an international, multi-disciplinary consensus development conference was held in the United States to develop a new classification system to apply to all forms of female sexual dysfunction regardless of etiology (International Consensus Development Conference on Female Sexual Dysfunctions: Definitions and Classifications, in press). This panel recommended maintaining four major categories of dysfunction (desire disorders, arousal disorder, orgasmic disorder, and sexual pain disorders), as described in the DSM-IV and ICD-10 (International Classification of Diseases) (World Health Organization, 1992)."	Inadequate	N
HADS [81]^a^	“depression subscale were largely based on the anhedonic state (…) psychic manifestations of anxiety neurosis”	Unclear	Inadequate	N
K6 [82]	"Non-specific psychological distress"	"The conceptualization of this task relied importantly on the work of Dohrenwend and his colleagues (Dohrenwend et al. 1980; Link & Dohrenwend, 1980). Their review of screening scales of nonspecific psychological distress showed that these scales typically include questions about a heterogeneous set of cognitive, behavioural, emotional and psychophysiological symptoms that are elevated among people with a wide range of different mental disorders."	Inadequate	N
PAS [83]	"We previously defined adaptation as the dynamic and multidimensional process of coming to terms with the implications of a health threat and the outcomes of that process (…) the cognitive and emotional outcomes of coping. We strived to select key components of adaptation, thereby simplifying a complex outcome."	"Both the Transactional Theory of Stress and Coping [3] and the Cognitive Theory of Adaptation [6] were used to guide the selection of four domains of adaptation included in the PAS. The domains are coping efficacy, self-esteem, spiritual/existential well-being, and social integration. Coping appears as a key mediator of adaptation in both theories and informed our choice of coping efficacy as the first domain. Taylor’s theory further elucidates a key role for selfesteem, 'meaning making' that leads to existential well-being, and re-engagement in social encounters."	Inadequate	N
PedsQL FIM [84]	Unclear	"A multidimensional instrument that could stand alone, or be easily integrated into the PedsQL™ Measurement Model [10]. The PedsQL™ Measurement Model includes not only generic health-related quality of life [11–13] and disease-specific measurement instruments [14–18], but also generic measures of fatigue [15, 19], healthcare satisfaction [20, 21] and evaluations of the healthcare built environment [21]."	Inadequate	N
PPC [51, 85]	"Perceived personal control is 'the belief that one has at one’s disposal a response that can influence the aversiveness of an event' [Thompson, 1981]."	"Lazarus and Folkman’s Transactional Model of Stress and Coping [Folkman, 1984] theorizes that the perception of stress depends on a number of subjective, cognitive judgments that arise from the dynamic interaction of a person and his or her environment (…) We used a stress and coping perspective to understand the relationships among parental uncertainty, perceived control, and the contribution of the genetic counselor to learn about the influences of the health care provider within situations of uncertainty."	Inadequate	N
PSQI [86]	"'Sleep quality' includes quantitative aspects of sleep, such as sleep duration, sleep latency, or number of arousals, as well as more purely subjective aspects, such as 'depth' or 'restfulness' of sleep."	Unclear	Inadequate	N
PSS [87, 88]	"The degree to which situations in one's life are appraised as stressful (…) designed to tap the degree to which respondents found their lives unpredictable, uncontrollable, and overloading"	"It is a common assumption among health researchers that the impact of 'objectively' stressful events is, to some degree, determined by one's perceptions of their stressfulness, e.g., see Lazarus (1966, 1977) (…) This implication is counter to the view that persons actively interact with their environments, appraising potentially threatening or challenging events in the light of available coping resources (Lazarus, 1966, 1977). From this latter perspective, stressor effects are assumed to occur only when both (a) the situation is appraised as threatening or otherwise demanding and (b) insufficient resources are available to cope with the situation."	Inadequate	N
QRS [89, 90]	"Burden imposed on the family [and] the family's emotional response to that burden"	"Family stress is a product of innumerable variables: degree of handicap or illness, personal resources of family members, financial resources, community support, and so forth. In addition to identifying the relevant variables that contribute to or ameliorate stress in families caring for ill or disabled family members, it is important to measure the family's response to those stressors"	Inadequate	N
SCL-90-R [91, 92]	"Symptomatology and psychological distress"	Unclear	Inadequate	N
SF-12 [93, 94]	"Health status"	Unclear	Inadequate	N
SF-36 [95–98]^a^	“‘Health’, eight concepts: physical functioning, social and role functioning, mental health, general health perceptions, bodily pain, and vitality.”	“The eight health concepts were selected from 40 concepts included in the Medical Outcomes Study (MOS). Those chosen represent the most frequently measured concepts in widely used health surveys and those most affected by disease and treatment.^68,70^ SF-36 items also represent multiple operational indicators of health, including behavioral function and dysfunction, distress and well-being, objective reports and subjective ratings, and both favorable and unfavorable self-evaluations of general health status.^68^”	Inadequate	N
SNQ [79]	Unclear	Unclear	Inadequate	N
STAI-X [99]	"State anxiety (A-State) is conceptualized as a transitory emotional state or condition of the human organism that is characterized by subjective, consciously perceived feelings of tension and apprehension, and heightened autonomic nervous system activity. A-States may vary in intensity and fluctuate over time. Trait anxiety (A-Trait) refers to relatively stable individual differences in anxiety proneness, that is, to differences between people in the tendency to respond to situations perceived as threatening with elevations in A-State intensity."	"The conceptions of trait and state anxiety that guided the construction of the STAI are considered in greater detail by Spielberger (1966a)"	Inadequate	N
SWLS [100]^a^	“Life satisfaction refers to a cognitive, judgmental process. Shin and Johnson (1978) define life satisfaction as ‘a global assessment of a person’s quality of life according to his chosen criteria’ (p. 478)”	Unclear	Inadequate	N
WHOQOL-BREF [101–104]^a^	“It is a broad ranging concept incorporating, in a complex way, the person’s physical health, psychological state, level of independence, social relationships, personal beliefs, and relationship to salient features of the environment (…) At minimum, quality of life includes the following dimensions: physical (individuals’ perception of their physical state), psychological (individuals’ perception of their cognitive and affective state) and social (individuals’ perception of the interpersonal relationship relationships and social roles in their life). (…) The WHOQOL includes a spiritual dimension (the person’s perception of ‘meaning in life’, or the overarching personal beliefs that structure and qualify experience).”	“An initial step involved achieving consensus on a working definition of quality of life as a person's perception of his/her position in life within the context of the culture and value systems in which he/she lives and in relation to his/her goals, expectations, standards, and concerns (…) This definition highlights the group's commitment to an essentially subjective concept that encompasses the multidimensional nature of quality of life (physical, psychological, social, etc.).”	Doubtful	Y
WAC-DBMD [51]	"Designed to assess amount, frequency, and intensity of DBMD-specific care worry"	Unclear	Inadequate	N
ZBI (4 item) [105]	"Burden"	Unclear	Inadequate	N
ZBI (12 item) [105]	"Burden"	Unclear	Inadequate	N
ZBI (22 item) [106]	"Burden"	Unclear	Inadequate	N

Citations next to instrument name are instrument development papers

^a^Data already extracted and adapted from Powell et al. 2020, including additional reference(s) for development studies not identified in this review [20]

A total of 7 instruments featured some form of piloting/cognitive interviewing during their development (Care-related Quality of Life Instrument [CarerQoL], Caregiver Well-being Scale [CWBS], EQ-5D-5L, Family Problems Questionnaire [FPQ], Female Sexual Function Index [FSFI], QRS, State-Trait Anxiety Inventory form X [STAI-X]), during which participants were asked about the measure’s comprehensibility. Comprehensiveness was probed in 2 further instruments (CarerQoL, FPQ). Aside from the EQ-5D-5L, where comprehensibility was explored using a focus group methodology [61], the rest of the pilot studies either didn’t use qualitative methods or the reporting of the methods was poor. In short, there was little evidence of any robust qualitative methods in the development of these carer instruments.

Table 8 summarises the synthesised ratings for the content validity of the evaluated instruments, based on the available evidence and synthesised DMD carer reviewer ratings. Ratings are split into relevance, comprehensiveness, and comprehensibility. CarerQoL performed best in the ratings of instrument development. Carer ratings were mixed, with a lot of inconsistency. No one instrument received a positive rating across all aspects of content validity from reviewers. For carer ratings, the best performing instrument was the PedsQL Family Impact Module (PedsQL FIM). The joint worst performing instruments were the SWLS, 12-Item Short Form Survey (SF-12), FSFI, and Caregiver Strain Index Plus (CSI+). Overall, primarily due to a lack of evidence and inconsistent ratings across carers, the overall rating for the content validity of all instruments evaluated in this study was inconsistent. No studies were identified which had independently assessed the content validity of the QoL instruments in samples of carers of people with DMD. Contributing to the low quality of evidence observed.

**Table 8:** COSMIN ratings of the relevance, comprehensiveness, and comprehensibility of instruments used to assess carer quality of life in DMD

Instrument	Instrument development study			Reviewer ratings			Overall ratings			Quality of evidence
	Relevance	Comprehensiveness	Comprehensibility	Relevance	Comprehensiveness	Comprehensibility	Relevance	Comprehensiveness	Comprehensibility	
BDI	−	?	?	± (+)	−	+	±	−	+	Very low^a^
CarerQoL	±	+	+	±	±	±	±	±	±	Very low
CSI	±	?	?	±	± (+)	±	±	±	±	Very low
CSI+	−	?	?	±	±	−	±	±	-	Very low
CWBS	±	?	+	± (+)	± (−)	+	±	±	+	Very low
DUKE	±	?	?	± (+)	± (+)	± (+)	±	±	±	Very low
EQ-5D-3L	?	?	?	±	−	± (+)	±	−	±	Very low^a^
EQ-5D-5L	±	?	+	±	± (+)	± (+)	±	±	±	Low
ESS	±	?	?	±	± (−)	+	±	±	+	Very low
ESSI	±	?	?	±	± (−)	±	±	±	±	Very low
FAPGAR	±	?	?	±	± (−)	±	±	±	±	Very low
FPQ	±	?	?	±	±	±	±	±	±	Very low
FSFI	±	?	?	±	−	±	±	−	±	Very low
HADS	−	?	?	± (+)	−	+	±	−	+	Very low^a^
K6	±	?	?	+	±	± (+)	+	±	±	Very low
PAS	±	?	?	+	−	± (+)	+	−	±	Very low
PedsQL FIM	−	?	?	+	± (+)	+	±	±	+	Very low
PPC	±	?	?	+	±	± (+)	+	±	±	Very low
PSQI	±	?	?	±	± (−)	± (+)	±	±	±	Very low
PSS	±	?	?	± (+)	−	+	±	−	+	Very low
QRS	±	?	+	±	±	± (−)	±	±	±	Very low
SCL-90-R	±	?	?	±	± (−)	± (+)	±	±	±	Very low
SF-12	−	?	?	±	−	±	±	−	±	Very low
SF-36	?	?	?	±	± (−)	± (+)	±	±	±	Very low^a^
SNQ	−	?	?	± (+)	± (−)	+	±	±	+	Very low
STAI-X (state)	±	?	?	±	−	+	±	−	+	Very low
STAI-X (trait)	±	?	?	±	−	± (+)	±	−	±	Very low
SWLS	−	?	?	± (−)	−	± (+)	±	−	±	Very low^a^
WHOQOL-BREF	±	?	?	± (+)	± (-)	± (+)	±	±	±	Very low^a^
WAC-DBMD	−	?	?	± (+)	−	±	±	−	+	Very low
ZBI (4 item)	−	?	?	± (+)	−	± (+)	±	−	±	Very low
ZBI (12 item)	−	?	?	±	?	±	±	?	+	Very low
ZBI (22 item)	−	?	?	±	−	± (+)	±	−	±	Very low

+, satisfactory results; −, unsatisfactory results; ±, inconsistent results; ± (+), inconsistent results trending towards satisfactory; ± (−), inconsistent results trending towards unsatisfactory; ?, indeterminate

^a^Development study and quality of evidence based on ratings in Powell et al. [20]

#### Structural validity

Only one study had assessed the structural validity of an instrument evaluated in this review, the ZBI (22-item) [60]. Landfeldt et al. (2019) examined the structural validity of the ZBI (22-item) using a Rasch partial credit model in a study with a high quality of evidence and found this measurement property was unsatisfactory in DMD carers. The results are summarised in Table 9.

**Table 9:** Results of studies assessing structural validity of the instruments included in the review

Instrument	N	Mean Age (SD)	% female	Country	Analysis model	Key result(s)	Rating of measurement property	
							Rating	Quality of evidence
ZBI (22 item) [60]	475	44 (NR)	81	UK, US	Rasch partial credit model	"In total, nine of 22 items (41%) displayed model misfit in terms of estimated residuals, all but two at a significant χ2 probability. Four misfitting items had a large negative residual, suggesting that these may not add any new information to the scale. The overall item–trait interaction chi-square value was 499, 198 degrees of freedom, *p* < 0.001, indicating that the items were not working as expected across different levels (i.e., class intervals) of burden (…) Mean item dependency was low (0.042) (…) Disordered thresholds were identified for 13 of 22 items (59%) (…) Minimal floor effect (< 1%, 1 of 475) and no ceiling effect (…) Fit residuals ranged from 0.02 to 4.76 (9 items exhibited misfit) (…) Taken together, results from our analysis showed that the English (UK and US) version of ZBI may not be regarded as a unidimensional, interval rating scale of burden among caregivers to patients with DMD.”	−	High

Citation next to the instrument is for the study assessing this measurement property

NR, not reported

#### Internal consistency

Seven studies were identified which assessed the internal consistency of an instrument and/or its subscales. The results are summarised in Table 10. Most instruments evaluated demonstrated a satisfactory rating for internal consistency, with a moderate or high quality of evidence. Exceptions were the FPQ which was indeterminate as the Cronbach’s alpha value was reported as a range across all subscales, the QRS which had a low quality of evidence, and the Social Networks Questionnaire (SNQ) (subscale A) which received an unsatisfactory internal consistency rating.

**Table 10:** Results of studies assessing internal consistency of the instruments included in the review

Instrument	N (DMD subsample)	Mean (SD) Age (DMD subsample)	% Female (DMD subsample)	Country	Cronbach’s α	Rating of measurement property	
						Rating	Quality of evidence
CWBS [35]	60	NR	41.7	India	0.92	+	Moderate
DUKE [37]	126	43 (6.1)	57.4	Taiwan	0.81	+	High
FAPGAR [37]	126	43 (6.1)	57.4	Taiwan	0.89	+	High
FPQ [46]^a^	336 (246 DMD)	41.2 (6.2) for DMD carers	84.2 (83.3 DMD)	Italy	Ranged from 0.66 to 0.87	?	High
HADS (Anxiety) [32]	82 (71 DMD)	40.40 (6.98)	100	USA	0.87	+	Moderate
HADS (Depression) [32]	82 (71 DMD)	40.40 (6.98)	100	USA	0.8	+	Moderate
PAS [51]	205 at baseline (147 at year 1, 144 at year 2)	44 (8.7)	100	USA	0.96	+	High
PPC [51]	205 at baseline (147 at year 1, 144 at year 2)	44 (8.7)	100	USA	0.79	+	High
PSS [32]	82 (71 DMD)	40.40 (6.98)	100	USA	0.89	+	Moderate
QRS [53]	36	43.04 (5.52)	69.44	Canada	0.86	+	Low
SF-36 [32]^a^	82 (71 DMD)	40.40 (6.98)	100	USA	Ranged from 0.76 to 0.88	+	Moderate
SNQ (subscale A) [46]	336 (246 DMD)	41.2 (6.2) for DMD carers	84.2 (83.3 DMD)	Italy	0.69	−	High
WAC-DBMD [51]	205 at baseline (147 at year 1, 144 at year 2)	44 (8.7)	100	USA	0.89	+	High
ZBI (22 item) [60]	475	44 (NR)	81	UK, US	0.914	+	High
ZBI (12 item) [51]	205 at baseline (147 at year 1, 144 at year 2)	44 (8.7)	100	USA	0.89	+	High

Citation next to the instrument is for the study assessing this measurement property

NR, not reported

^a^FPQ and SF-36 subscales were assessed separately, however Cronbach’s α was reported as a range across all scales

#### Hypotheses testing for construct validity

Table 11 summarises the results of studies with evidence on the construct validity of the instruments included in the review. Evidence on construct validity was observed for 30 instruments/instrument subscales, from a total of 19 studies, featuring a mixture of convergent (i.e. correlational) and known groups validity. Performance of the instruments against reviewer a priori defined hypotheses was mixed and the quality of evidence ranged from very low to high. Some instruments, such as the WHOQOL-BREF and some SF-36 subscales, performed inconsistently with a moderate or high quality of evidence. Others, such as the Family APGAR (FAPGAR), HADS and other SF-36 subscales, performed well with a high quality of evidence.

**Table 11:** Results of studies assessing construct validity of the instruments included in the review

Instrument	Validity study type	N (DMD subsample)	Mean (SD) Age (DMD subsample)	% Female (DMD subsample)	Country	Results consistent with reviewer hypotheses	Rating	Quality of evidence
CSI	Known groups [7]	80	57 (6.8)	69	Netherlands	0 out of 1	−	Very low
DUKE	Convergent [36]	126	43 (6.1)	57.14	Taiwan	3 out of 4	+	High
	Known groups [40]	113 (55 DMD)	45.89 (7.27) (DMD 44.87 (7.23))	57.52 (DMD 56.36)	Taiwan	0 out of 1	−	
EQ-5D-3L	Known groups [31]	770	44 (8)	79	Germany, Italy, UK, US	13 out of 18	−	Low
ESS	Known groups [45]	70 (35 DMD)	46.3 (1.3) for DMD carers	100	Brazil	1 out of 2	−	Low
FAPGAR	Convergent [36]	126	43 (6.1)	57.14	Taiwan	3 out of 4	+	High
	Known groups [40]	113 (55 DMD)	45.89 (7.27) (DMD 44.87 (7.23))	57.52 (DMD 56.36)	Taiwan	1 out of 1	+	
HADS (Anxiety)	Convergent [7]	80	57 (6.8)	69	Netherlands	2 out of 2	+	High
	Known groups [32]	82 (71 DMD)	40.40 (6.98)	100	USA	1 out of 1	+	
HADS (Depression)	Convergent [7]	80	57 (6.8)	69	Netherlands	1 out of 1	+	High
	Known groups [32]	82 (71 DMD)	40.40 (6.98)	100	USA	1 out of 1	+	
PAS	Convergent [51]	205 at baseline (147 at year 1, 144 at year 2)	44 (8.7)	100	USA	3 out of 8	−	Very low
PedsQL FIM	Known groups [49]	15	41.7 (not reported)	60	China	1 out of 2	−	Very low
PSQI	Known groups [52]	64 (32 DMD)	46.2 (8.1) for DMD carers	100	Brazil	1 out of 1	+	Moderate
	Known groups [45]	70 (35 DMD)	46.3 (1.3) for DMD carers	100	Brazil	0 out of 1	−	
PSS	Known groups [32]	82 (71 DMD)	40.40 (6.98)	100	USA	1 out of 1	+	Low
QRS	Convergent [53]	36	43.04 (5.52)	69.44	Canada	3 out of 3	+	Moderate
	Convergent [54]	56 (17 DMD)	43.4 (4.5) for DMD carers	64.29 (82.35 for DMD)	Canada	1 out of 1	+	
SCL-90-R	Convergent [56]	35	NR	91.43	USA	6 out of 8	+	Very low
SF-12 (MCS)	Known groups [31]	770	44 (8)	79	Germany, Italy, UK, US	2 out of 3	−	Low
SF-36 (BP/Pain)	Convergent + known groups [33]	62 (53 DMD)	40.1 (8.8) for DMD carers	66.13 (62.2 DMD)	Taiwan	5 out of 8	−	High
	Known groups [32]	82 (71 DMD)	40.40 (6.98)	100	USA	1 out of 1	+	
SF-36 (E|F/VT)	Convergent + known groups [33]	62 (53 DMD)	40.1 (8.8) for DMD carers	66.13 (62.2 DMD)	Taiwan	5 out of 8	−	High
	Known groups [32]	82 (71 DMD)	40.40 (6.98)	100	USA	1 out of 1	+	
SF-36 (EW/MH)	Convergent + known groups [33]	62 (53 DMD)	40.1 (8.8) for DMD carers	66.13 (62.2 DMD)	Taiwan	6 out of 8	+	High
	Known groups [32]	82 (71 DMD)	40.40 (6.98)	100	USA	1 out of 1	+	
SF-36 (GH)	Convergent + known groups [33]	62 (53 DMD)	40.1 (8.8) for DMD carers	66.13 (62.2 DMD)	Taiwan	6 out of 8	+	High
	Known groups [32]	82 (71 DMD)	40.40 (6.98)	100	USA	1 out of 1	+	
SF-36 (MCS)	Convergent [33]	62 (53 DMD)	40.1 (8.8) for DMD carers	66.13 (62.2 DMD)	Taiwan	2 out of 4	−	Low
SF-36 (PCS)	Convergent [33]	62 (53 DMD)	40.1 (8.8) for DMD carers	66.13 (62.2 DMD)	Taiwan	4 out of 4	+	Low
SF-36 (PF)	Convergent + known groups [33]	62 (53 DMD)	40.1 (8.8) for DMD carers	66.13 (62.2 DMD)	Taiwan	8 out of 8	+	High
	Known groups [32]	82 (71 DMD)	40.40 (6.98)	100	USA	1 out of 1	+	
SF-36 (RE)	Convergent + known groups [33]	62 (53 DMD)	40.1 (8.8) for DMD carers	66.13 (62.2 DMD)	Taiwan	8 out of 8	+	High
	Known groups [32]	82 (71 DMD)	40.40 (6.98)	100	USA	1 out of 1	+	
SF-36 (RP)	Convergent + known groups [33]	62 (53 DMD)	40.1 (8.8) for DMD carers	66.13 (62.2 DMD)	Taiwan	7 out of 8	+	High
	Known groups [32]	82 (71 DMD)	40.40 (6.98)	100	USA	1 out of 1	+	
SF-36 (SF)	Convergent + known groups [33]	62 (53 DMD)	40.1 (8.8) for DMD carers	66.13 (62.2 DMD)	Taiwan	4 out of 8	−	High
	Known groups [32]	82 (71 DMD)	40.40 (6.98)	100	USA	1 out of 1	+	
SNQ (Subscale A)	Convergent [46]	336 (246 DMD)	41.2 (6.2) for DMD carers	84.2 (83.3 DMD)	Italy	1 out of 3	−	Very low
STAI-X (state)	Known groups [55]	37 (17 DMD)	37.68 (8) for DMD carers	100	Turkey	0 out of 1	−	Very low
STAI-X (trait)	Known groups [55]	37 (17 DMD)	37.68 (8) for DMD carers	100	Turkey	0 out of 1	−	Very low
SWLS	Convergent [54]	56 (17 DMD)	43.4 (4.5) for DMD carers	64.29 (82.35 for DMD)	Canada	1 out of 1	+	Very low
WHOQOL-BREF	Convergent [58]	31	38 (not reported)	83.87	Brazil	1 out of 3	−	Moderate
	Convergent + known groups [57]	30	39.20 (8.32)	93.3	Brazil	5 out of 6	+	
	Known groups [34]	90 (67 DMD)	42.9 (8.7)	90	South Korea	0 out of 1	−	
ZBI (22 item)	Convergent [58]	31	38 (not reported)	83.87	Brazil	2 out of 3	−	Low
	Convergent [59]	35	38.7 (8.2)	91.4	Brazil	0 out of 4	−	
	Known groups [31]	770	44 (8)	79	Germany, Italy, UK, US	3 out of 3	+	

Citation next to validity study type is for the study assessing this measurement property.

BP/Pain, bodily pain/pain subscale; E|F/VT, energy|fatigue/vitality subscale; EW/MH, emotional wellbeing/mental health subscale; GH, general health subscale; MCS, mental component summary score; PCS, physical component summary score; PF, physical functioning subscale; RE, role emotional subscale; RP, role physical subscale; SF, social functioning subscale

#### Cross-cultural validity/measurement invariance

Landfeldt et al. was the only study identified in the review that evaluated the measurement invariance of an included instrument, the ZBI (22 item), using differential item functioning [60]. Measurement invariance was observed (i.e. no differential item functioning) using the criteria adopted in the study, giving the ZBI (22 item) a satisfactory rating on that measurement property. However, this was based on a very low quality of evidence, as it was doubtful that groups were similar except for the grouping variable and the group sample sizes were lower than recommended by COSMIN. The results are summarised in Table 12.

**Table 12:** Results of studies assessing responsiveness of the instruments included in the review

Instrument	N	Mean (SD) Age	% Female	Country	Results consistent with reviewer hypotheses	COSMIN rating	Quality of evidence
PAS [51]	205 at baseline (147 at year 1, 144 at year 2)	44 (8.7)	100	USA	3 out of 3	+	Moderate
PPC [51]	205 at baseline (147 at year 1, 144 at year 2)	44 (8.7)	100	USA	2 out of 3	−	Moderate
WAC-DBMD [51]	205 at baseline (147 at year 1, 144 at year 2)	44 (8.7)	100	USA	1 out of 1	+	Moderate
ZBI (12 item) [51]	205 at baseline (147 at year 1, 144 at year 2)	44 (8.7)	100	USA	0 out of 3	−	Moderate

Citation next to instrument is for the study assessing this measurement property

#### Responsiveness

One study, with a moderate quality of evidence, was identified which assessed responsiveness of four of the instruments included in this review in carers of people with DMD [51]. The results are summarised in Table 13. Both the Psychological Adaptation Scale (PAS) and Worry about Care for Child with DBMD (WAC-DBMD) received satisfactory ratings, and the Perceived Personal Control Questionnaire (PPC) and ZBI (12 item) received unsatisfactory ratings, based on reviewers’ a priori hypotheses.

**Table 13:** Results of studies assessing measurement invariance of the instruments included in the review

Instrument	N	Mean Age (SD)	% female	Country	Analysis model	Key result(s)	Rating of measurement property	
							Rating	Quality of evidence
ZBI [60]	475	44 (NR)	81	UK, US	ANOVA (DIF)	“Analysis of scale stability showed that there was no significant uniform differential item functioning (i.e., a systematic difference across the full range of level of burden) or nonuniform differential item functioning (i.e., nonuniformity in the differences across level of burden) by country (UK vs. US; *p* > 0.002 and *p* > 0.009) or by sex (female vs. male; *p* > 0.004 and *p* > 0.028).”	+	Very low

Citation next to the instrument is for the study assessing this measurement property

DIF, differential item functioning; NR, not reported

#### Other measurement properties

No studies were found that contained evidence on the reliability, measurement error, or criterion validity of any of the instruments included in this review.

## Discussion

This systematic review was designed to identify instruments used to assess elements of QoL in informal carers of people with DMD and evaluate the published evidence on their measurement properties in this population. Overall, there was a picture of low quality or missing psychometric evidence across a variety of measurement properties for the instruments identified. The majority of the measures did not involve carers in their development and there were no content validity studies in DMD caregivers to assess their suitability (in terms of their relevance, comprehensiveness, and comprehensibility). This, combined with inadequate or doubtful instrument development studies by COSMIN standards, and mixed caregiver ratings of the instruments themselves, lead to inconsistent results for content validity, based on a low quality of evidence. Furthermore, only one study assessed the structural validity of an included instrument in DMD carers, revealing unsatisfactory results [60]. These two measurement properties (content and structural validity) are considered the most important in the COSMIN framework [13, 107, 108], and the finding that evidence on them is lacking and/or unsatisfactory for DMD caregivers is revealing. Instead, the questionnaires included in this review have been used in DMD studies by researchers without also assessing or confirming they are reliable and valid for use with DMD carers. For example, the ZBI (22 item) is one of the most popular tools used in DMD carers [31, 41, 42, 58–60], but has unsatisfactory measurement properties, including elements of content validity and structural validity. This ultimately puts the validity of the conclusions from studies using such instruments into question.

As no previous content validity studies on QoL instruments have been conducted with DMD carers, the ratings provided by carer team members in this review represent the first insight into how people asked to complete these instruments evaluate them, in terms of their relevance, comprehensiveness, and comprehensibility. This is a strength of the review. Incorporating consideration of the lived experience into the assessment of existing instruments not only adds to the validity of the findings of the review, but also highlighted some of the inadequacies of the questionnaires themselves. While conducted using COSMIN procedures, it should be acknowledged that this is a limited assessment of how DMD carers responded to a selection of the instruments included in this review. As the number of instruments was large, it was not possible to have the same carers rating all of the instruments, so individual differences in interpretation and rating are not held consistent. Further, ratings were completed individually and synthesised, not arrived at through consensus. Thus, this is not a full content validity study and further work is urgently needed, which would benefit from in-depth qualitative techniques. Nevertheless, this does provide the first, preliminary insight into how these instruments perform in the eyes of Duchenne carers. From this insight, the PedsQL FIM had the most potential as a QoL measure for Duchenne carers.

Aside from content and structural validity, internal consistency was a measurement property that was quite frequently reported, often with satisfactory results (with the exception of SNQ and FPQ). Mixed results were observed on construct validity, but it should be acknowledged that evaluation of this psychometric property is determined by a priori reviewer-generated hypotheses and expectations about how QoL instruments and known-group criteria should be related [15]. The evidence differs across all studies (i.e. in terms of what a QoL instrument is chosen to be compared to by researchers) and thus not all instruments are subjected to the same test of validity. There were also only a handful of studies on measurement invariance and responsiveness. While some measures performed well on these criteria, it is our view that these should not be used to advocate the use of an instrument in the absence of good content and structural validity, the two most important measurement properties [13].

An aim of this review was to make a recommendation for which questionnaire(s) (if any) are best suited to assess QoL in DMD informal caregivers. Making such a recommendation is difficult as there was no instrument with evidence that excelled across all measurement properties (or even across the foundational measurement properties of content and structural validity) and the quality of available evidence was often low. Further, many of the instruments identified in this review were designed/used to assess only one aspect of carer QoL, rather than QoL as a whole. CarerQoL performed best in terms of instrument development, but was inconsistent in carer reviewer ratings, and had no additional evidence on its psychometric properties. PedsQL FIM received the best ratings from carer reviewers and while the instrument received an unsatisfactory rating for construct validity, this was based on a very low quality of evidence. Our recommendation is thus, first and foremost, for additional high-quality research into the measurement properties of instruments included in this review in Duchenne caregivers. In the interim, we recommend that the PedsQL FIM is considered for future use and evaluation as a multidimensional QoL instrument that appears to be received well by Duchenne caregivers.

It was of interest to note that during the sifting process of literature as part of this review, that a number of qualitative studies exploring the impact of caring for individuals with DMD were identified (e.g. [109–111]). Furthermore, work is continuing to emerge in this area [112]. Whilst these were not selected for inclusion within this review (due to predetermined inclusion criteria), it is clear that there is a body of evidence on this important topic. Consideration, and potential synthesis, of such studies could be a meaningful area of future study. It is possible that existing qualitative literature highlights aspects of carer QoL that are not captured when measuring QoL using any of the instruments identified in this review. Furthermore, existing qualitative literature may also identify any potential cultural and/or country differences which may be important. Given that DMD is a rare condition, large-scale prospective studies of carer QoL can only be achieved using a multi-country recruitment approach. Ensuring that any instrument used to measure carer QoL is culturally appropriate will be necessary.

The focus of this review was to report on the measurement properties of instruments that have been used to quantify QoL of carers of individuals with DMD. However, it is clear when applying COSMIN methodology that the content validity of the instruments identified was questionable. It could be argued that the appropriateness of such questionnaires to assess carer QoL for other health conditions is not justified. Whilst there is still a requirement to assess the relevance, comprehensiveness and comprehensibility of the instruments for other health conditions, this does not overcome the limited evidence for the content validity (i.e. development) of the measures themselves. This review has highlighted the need for future studies to support the content validity of instruments for the target population. It can be postulated that other neuromuscular disorders could imply similar impacts upon carer QoL, however this has not been explored within the context of this review. Furthermore, there are other instruments available which can be used to measure carer QoL which were not included in this review (as they had not been used in studies relating to DMD).

This review is not without its limitations. Whilst the methodological approach adopted is recognised and robust, it does have some limitations, as previously noted [16, 20]. Firstly, the COSMIN appraisal tools assume a worst score counts system. If a study fails to report key details, this results in a reduced rating of the instrument to *doubtful* or *inadequate*. Secondly, many of the questionnaires identified in the review could be considered as legacy measures. They were developed at a time when detailed descriptions of instrument development were not necessarily reported and/or different methods for instrument design were accepted. The COSMIN approach is such that these instruments score poorly. It is important to recognise that this does not necessarily mean that the development of these instruments was fundamentally flawed or inappropriate such that have no utility whatsoever, but that an assessment of the *available evidence* by modern standards has found them lacking. Thirdly, whilst the inclusion of the lived experience (i.e. carer perspective) was incorporated into this review, it must be acknowledged that there may be a degree of bias associated with the responses. Whilst efforts were made to mitigate this (by providing average ratings, i.e. obtaining more than one carer ratings per instrument), it must be noted that all respondents were from the UK. It is possible that their experiences (and their experiences of the UK health and social care systems) may have influenced their ratings. It is not clear whether the findings are replicable in other countries. In addition, the vast majority of informal carer ratings were provided from mothers. Indeed, a number of the studies included in the review also assessed the impact of mother’s QoL (presumably with the assumption that mothers are usually the primary caregiver). However, it can be argued that modern-day parenting situations and roles have altered over recent years, and it cannot be assumed that paternal ratings of the instruments included in the review would align with maternal views.

Due to the large number of instruments included within our review, and the focus on quality of life as a multidimensional construct, we made a pragmatic decision to present the results of instruments, rather than individual subscales (where appropriate). Furthermore, in the assessment of comprehensiveness of the instrument, we applied this to the construct of overall quality of life (rather than the construct the instrument may have been designed to measure).Finally, the assessment of measurement properties of the instruments undertaken here does not incorporate consideration of the acceptability or feasibility of the identified measures. These are also important factors when assessing their suitability. For example, this may include the length of the instrument and how cognitively demanding it is. Practical issues of PROM availability, such as costs and licensing requirements, availability in all required languages, and mode of administration (i.e. electronic versus paper) also play a key role. This review was limited to those instruments where a free or review copy was available for research purposes.

## Conclusion

The instruments used to measure impact on Duchenne carer quality of life have limited psychometric evidence to support their use. To that end, the published evidence reporting QoL in carers of people with DMD may not accurately reflect the true impact of caregiving on QoL. Further work is thus required to investigate the measurement properties of common QoL measures in DMD carers, including content validity studies. Research should also examine whether a) the constructs of the instruments identified as part of this review map onto a conceptual framework of carer quality of life in DMD; and b) whether this differs for other paediatric life-limiting conditions. Given the results of this review, work may also be justified in the development of condition-specific carer QoL measures (or within paediatric life-limiting conditions) for use in DMD to better capture the true impacts of the condition on carers. In the interim, we recommend the consideration of the PedsQL FIM as a QoL measure in Duchenne carers, as it showed most promise from evaluation by carers themselves.

## Supplementary Information

**Additional file 1:** Database search strategies

**Additional file 2:** Data extracted from included studies

**Additional file 3:** Instructions for carer ratings of instruments’ content validity

**Additional file 4:** Full list of questionnaires identified in the searches

**Additional file 5:** COSMIN rating sheets

## Data Availability

All data generated or analysed during this study are included in this published article [and its supplementary information files].

## Abbreviations

BDI: Beck Depression Inventory
CarerQoL: Care-related Quality of Life Instrument
COSMIN: COnsensus-based Standards for the selection of health Measurement INstruments
CSI: Caregiver Strain Index
CSI +: Caregiver Strain Index Plus
CWBS: Carer Well-being Scale
DIF: Differential item functioning
DMD: Duchenne muscular dystrophy
DUKE: DUKE Health Profile
EQ-5D-3L: EuroQoL 5-domain 3-Level
EQ-5D-5L: EuroQoL 5-domain 5-Level
ESS: Epworth Sleepiness Scale
ESSI: ENRICHD Social Support Instrument
FAPGAR: Family APGAR (Adaptability, Partnership, Growth, Affection, and Resolve)
FPQ: Family Problems Questionnaire
FSFI: Female Sexual Function Index
GRADE: Grading of Recommendations Assessment, Development and Evaluation
HADS: Hospital Anxiety and Depression Scale
HTA: Health technology assessment
K6: Kessler Psychological Distress Scale
NICE: National Institute for Health and Care Excellence
PAS: Psychological Adaptation Scale
PedsQL FIM: PedsQL Family Impact Module
PPC: Perceived Personal Control Questionnaire
PPIE: Patient and public involvement and engagement
PRISMA: Preferred Reporting Items for Systematic Reviews and Meta-Analyses
PROM: Patient reported outcome measure
PROSPERO: International Prospective Register of Systematic Reviews
PSQI: Pittsburgh Sleep Quality Index
PSS: Perceived Stress Scale
QALY: Quality-adjusted life year
QRS: Questionnaire on Resources and Stress
QoL: Quality of life
SCL-90-R: Symptom Checklist-90-Revised
SF-12: 12-Item Short-Form Health Survey
SF-12 (MCS): 12-Item Short-Form Health Survey (Mental Component Score subscale)
SF-36: 36-Item Short-Form Health Survey
SF-36 BP/Pain: 36-Item Short-Form Health Survey (Bodily Pain/Pain subscale)
SF-36 E|F/VT: 36-Item Short-Form Health Survey (Energy|Fatigue/Vitality subscale)
SF-36 EW/MH: 36-Item Short-Form Health Survey (Emotional Wellbeing/Mental Health subscale)
SF-36 GH: 36-Item Short-Form Health Survey (General Health subscale)
SF-36 MCS: 36-Item Short-Form Health Survey (Mental Component Score subscale)
SF-36 PCS: 36-Item Short-Form Health Survey (Physical Component Score subscale)
SF-36 PF: 36-Item Short-Form Health Survey (Physical Functioning subscale)
SF-36 RE: 36-Item Short-Form Health Survey (Emotional Role Functioning subscale)
SF-36 RP: 36-Item Short-Form Health Survey (Physical Role Functioning subscale)
SF-36 SF: 36-Item Short-Form Health Survey (Social Functioning subscale)
SNQ: Social Networks Questionnaire
STAI-X: State-Trait Anxiety Inventory (form X)
SWLS: Satisfaction with Life Scale
WHOQOL-BREF: World Health Organisation Quality of Life Scale-Brief Version
WAC-DBMD: Worry about Care for Child with Duchenne and Becker Muscular Dystrophy
ZBI: Zarit Burden Interview

## Footnotes

1 As per COSMIN guidance, when the quality of an instrument’s development has already been evaluated it does not need to be rated again [12]. Accordingly, six instruments were not searched in Google Scholar, where development ratings were available from a previous review [20].

2 Study selection was carried out on and documented in Microsoft Excel, rather than a combination of EndNote and Microsoft Word as advised in the protocol.

3 We applied these rules to the additional possible rating outcomes ± ( +) and ± ( −), so that if ratings did not meet the criteria for an overall + or – rating for relevance, comprehensiveness, or comprehensibility they could still receive an overall ± (+) or ± (−) rating if the synthesised ratings were a mixture of + and ± (+) or – and ± (−), respectively.

4 Cohen’s d was calculated using an online calculator, available at https://lbecker.uccs.edu/. Where necessary SE was converted to SD using a formula in https://training.cochrane.org/handbook/current/chapter-06.
