# Supplementary material for: Measuring carer quality of life in Duchenne muscular dystrophy: a systematic review of the reliability and validity of self-report instruments using COSMIN
Source: Health Qual Life Outcomes. 2022 Apr 2;20:57. doi: 10.1186/s12955-022-01964-4 (PMC8977045; doi:10.1186/s12955-022-01964-4)
Supplement: Supplementary file 1 — Additional file 1: Database search strategies [file 12955_2022_1964_MOESM1_ESM.docx]

Article title: Measuring Carer Quality of Life in Duchenne Muscular Dystrophy: A Systematic Review of the Reliability and Validity of Self-Report Instruments Using COSMIN

Journal name: Journal of Neurology

Author names: Jill Carlton, Philip A. Powell, Ruth Wong, Project HERCULES Carer Group

Corresponding author: Jill Carlton, School of Health and Related Research (ScHARR), University of Sheffield, Regent Court, 30 Regent Street, Sheffield, S1 4DA, United Kingdom, [j.carlton@sheffield.ac.uk](mailto:j.carlton@sheffield.ac.uk), +44 114 222 0779

**Online Resource 1 – Database Search Strategies**

Two searches were undertaken in July and November 2020:

The following databases were searched:

1. MEDLINE (Ovid, 1946 to 2020)
2. Embase (Ovid, 1974 to 2020)
3. Cochrane Library
   1. Cochrane Database of Systematic Reviews (1996 to 2020)
   2. Cochrane Central Register of Controlled Trials (1898 to 2020)
4. Cumulative Index to Nursing & Allied Health (EBSCO, 1974 to 2020)
5. PsycINFO (Ovid, 1806 to 2020)
6. **STAGE 1 SEARCH (July 2020)**

| **Host** | **Database** | **Dates covered** | **Results** |
| --- | --- | --- | --- |
| Ovid | MEDLINE | 1946 to Present | 342 |
| Ovid | Embase | 1974 to Present | 765 |
| Wiley | Cochrane Database of Systematic Reviews | CDSR 1996- | 7 |
| Wiley | Cochrane Central Register of Controlled Trials | CENTRAL 1898- | 80 |
| EBSCO | CINAHL | 1974 to Present | 217 |
| Ovid | PsycINFO | 1806 to Present | 120 |
|  | Total retrieved | - | 1531 |
|  | **Unique records in database** | **-** | **978** |

**PROMS filter source:**

<https://cosmin.nl/wp-content/uploads/prom-search-filter-oxford-2010.pdf> [Accessed July 2020]

**Ovid MEDLINE(R) and Epub Ahead of Print, In-Process & Other Non-Indexed Citations, Daily and Versions(R) 1946 to July 07, 2020**

8^th^ July 2020

342 records

| **#** | **Searches** | **Results** |
| --- | --- | --- |
| 1 | Muscular Dystrophy/ | 13735 |
| 2 | duchenne*.mp. | 12784 |
| 3 | exp caregivers/ or (spillover* or spill over* or carer or carers or caregiver* or caring or "care giv*" or caregiving or family or families or partner* or husband or wife or wives or spouse or spouses or spousal or child or children or son* or daughter* or offspring* or dependent* or parent* or mother* or maternal or father* or paternal or sibling* or brother* or sister* or grandparent* or grandmother* or grandfather* or close person* or relative* or "next of kin" or kinship).tw. | 5369275 |
| 4 | (1 or 2) and 3 | 5483 |
| 5 | (HR-PRO or HRPRO or HRQL or HRQoL or QL or QoL).ti,ab. or quality of life.mp. or (health index* or health indices or health profile*).ti,ab. or health status.mp. or ((patient or self or child or parent or carer or proxy) adj (appraisal* or appraised or report or reported or reporting or rated or rating* or based or assessed or assessment*)).ti,ab. or ((disability or function or functional or functions or subjective or utility or utilities or wellbeing or well being) adj2 (index or indices or instrument or instruments or measure or measures or questionnaire* or profile or profiles or scale or scales or score or scores or status or survey or surveys)).ti,ab. | 748711 |
| 6 | (world health organization quality of life or whoqol).mp. | 3480 |
| 7 | (euro qual or euro qual5d or euro qol5d or eq-5d or eq5-d or eq5d or euroqual or euroqol or euroqual5d or euroqol5d).mp. | 11097 |
| 8 | (zarit caregiver burden interview or zarit burden interview or zbi).mp. | 709 |
| 9 | (pittsburgh sleep quality index or psqi).mp. | 4917 |
| 10 | (child health questionnaire parent form or child health questionnaire parent-form or chq-pf50).mp. | 166 |
| 11 | (sf12 or sf 12 or sf twelve or sftwelve).mp. | 4780 |
| 12 | (family problems questionnaire or fpq).mp. | 102 |
| 13 | (muscular dystrophy care schedule or md-cs or mdcs).mp. | 1350 |
| 14 | (female sexual function index or fsfi).mp. | 1934 |
| 15 | (caregiver strain index or csi).mp. | 3621 |
| 16 | (self-rated burden or srb).mp. | 2891 |
| 17 | (care-related quality of life instrument or carerqol).mp. | 44 |
| 18 | (family strain questionnaire or fsq).mp. | 148 |
| 19 | (family burden assessment scale or fbas).mp. | 89 |
| 20 | (cope inventory or coping with problems experienced or coping orientation to problems experienced or brief-cope or brief cope).mp. | 588 |
| 21 | (caregiver well-being scale or cwbs).mp. | 30 |
| 22 | (state-trait anxiety inventory or stai).mp. | 6157 |
| 23 | or/6-22 | 40920 |
| 24 | 4 and 5 | 329 |
| 25 | (1 or 2) and 23 | 25 |
| 26 | 24 or 25 | 342 |

**Embase 1974 to 2020 July 07**

8^th^ July 2020

765 records

| **#** | **Searches** | **Results** |
| --- | --- | --- |
| 1 | muscular dystrophy/ | 15485 |
| 2 | duchenne*.mp. | 20068 |
| 3 | exp caregiver/ or (spillover* or spill over* or carer or carers or caregiver* or caring or "care giv*" or caregiving or family or families or partner* or husband or wife or wives or spouse or spouses or spousal or child or children or son* or daughter* or offspring* or dependent* or parent* or mother* or maternal or father* or paternal or sibling* or brother* or sister* or grandparent* or grandmother* or grandfather* or close person* or relative* or "next of kin" or kinship).tw. | 6626428 |
| 4 | (1 or 2) and 3 | 8977 |
| 5 | (HR-PRO or HRPRO or HRQL or HRQoL or QL or QoL).ti,ab. or quality of life.mp. or (health index* or health indices or health profile*).ti,ab. or health status.mp. or ((patient or self or child or parent or carer or proxy) adj (appraisal* or appraised or report or reported or reporting or rated or rating* or based or assessed or assessment*)).ti,ab. or ((disability or function or functional or functions or subjective or utility or utilities or wellbeing or well being) adj2 (index or indices or instrument or instruments or measure or measures or questionnaire* or profile or profiles or scale or scales or score or scores or status or survey or surveys)).ti,ab. | 1121839 |
| 6 | (world health organization quality of life or whoqol).mp. | 5256 |
| 7 | (euro qual or euro qual5d or euro qol5d or eq-5d or eq5-d or eq5d or euroqual or euroqol or euroqual5d or euroqol5d).mp. | 20880 |
| 8 | (zarit caregiver burden interview or zarit burden interview or zbi).mp. | 1231 |
| 9 | (pittsburgh sleep quality index or psqi).mp. | 11209 |
| 10 | (child health questionnaire parent form or child health questionnaire parent-form or chq-pf50).mp. | 266 |
| 11 | (sf12 or sf 12 or sf twelve or sftwelve).mp. | 8198 |
| 12 | (family problems questionnaire or fpq).mp. | 133 |
| 13 | (muscular dystrophy care schedule or md-cs or mdcs).mp. | 2115 |
| 14 | (female sexual function index or fsfi).mp. | 4502 |
| 15 | (caregiver strain index or csi).mp. | 5853 |
| 16 | (self-rated burden or srb).mp. | 4162 |
| 17 | (care-related quality of life instrument or carerqol).mp. | 57 |
| 18 | (family strain questionnaire or fsq).mp. | 216 |
| 19 | (family burden assessment scale or fbas).mp. | 116 |
| 20 | (cope inventory or coping with problems experienced or coping orientation to problems experienced or brief-cope or brief cope).mp. | 1019 |
| 21 | (caregiver well-being scale or cwbs).mp. | 29 |
| 22 | (state-trait anxiety inventory or stai).mp. | 11757 |
| 23 | or/6-22 | 74536 |
| 24 | 4 and 5 | 733 |
| 25 | (1 or 2) and 23 | 61 |
| 26 | 24 or 25 | 765 |

**Cochrane Database of Systematic Reviews**

**Cochrane Central Register of Controlled Trials**

8^th^ July 2020

87 records

| **#** | **Searches** | **Results** |
| --- | --- | --- |
| #1 | MeSH descriptor: [Muscular Dystrophies] explode all trees | 444 |
| #2 | duchenne*:ti,ab,kw | 724 |
| #3 | MeSH descriptor: [Caregivers] explode all trees | 2051 |
| #4 | (spillover* or spill over* or carer or carers or caregiver* or caring or "care giv*" or caregiving or family or families or partner* or husband or wife or wives or spouse or spouses or spousal or child or children or son* or daughter* or offspring* or dependent* or parent* or mother* or maternal or father* or paternal or sibling* or brother* or sister* or grandparent* or grandmother* or grandfather* or close person* or relative* or "next of kin" or kinship):ti,ab,kw | 344509 |
| #5 | (#1 or #2) and (#3 or #4) | 441 |
| #6 | (HR-PRO or HRPRO or HRQL or HRQoL or QL or QoL or “quality of life” or “health index*” or “health indices” or “health profile*” or "health status"):ti,ab,kw | 116597 |
| #7 | ((patient or self or child or parent or carer or proxy) NEXT/1 (appraisal* or appraised or report or reported or reporting or rated or rating* or based or assessed or assessment*)):ti,ab,kw | 55635 |
| #8 | ((disability or function or functional or functions or subjective or utility or utilities or wellbeing or well being) NEXT/2 (index or indices or instrument or instruments or measure or measures or questionnaire* or profile or profiles or scale or scales or score or scores or status or survey or surveys)):ti,ab,kw | 35081 |
| #9 | [OR #6-#8] | 180154 |
| #10 | ("world health organization quality of life" or whoqol):ti,ab,kw | 1053 |
| #11 | ("euro qual" or "euro qual5d" or "euro qol5d" or "eq-5d" or "eq5-d" or "eq5d" or euroqual or euroqol or "euroqual5d" or "euroqol5d"):ti,ab,kw | 4256 |
| #12 | (“zarit caregiver burden interview” or ”zarit burden interview” or zbi):ti,ab,kw | 192 |
| #13 | (”pittsburgh sleep quality index” or psqi):ti,ab,kw | 2771 |
| #14 | (”child health questionnaire parent form” or ”child health questionnaire parent-form” or “chq-pf50”):ti,ab,kw | 22 |
| #15 | (“sf12” or ”sf 12” or ”sf twelve” or sftwelve):ti,ab,kw | 2100 |
| #16 | (”family problems questionnaire” or fpq):ti,ab,kw | 13 |
| #17 | (”muscular dystrophy care schedule” or md-cs or mdcs):ti,ab,kw | 28 |
| #18 | (”female sexual function index” or fsfi):ti,ab,kw | 851 |
| #19 | (”caregiver strain index” or csi):ti,ab,kw | 513 |
| #20 | (”self-rated burden” or srb):ti,ab,kw | 31 |
| #21 | (”care-related quality of life instrument” or carerqol):ti,ab,kw | 15 |
| #22 | (”family strain questionnaire” or fsq):ti,ab,kw | 39 |
| #23 | (”family burden assessment scale” or fbas):ti,ab,kw | 7 |
| #24 | (”cope inventory” or ”coping with problems experienced” or ”coping orientation to problems experienced” or ”brief-cope” or ”brief cope”):ti,ab,kw | 111 |
| #25 | (”caregiver well-being scale” or cwbs):ti,ab,kw | 1 |
| #26 | (”state-trait anxiety inventory” or stai):ti,ab,kw | 1666 |
| #27 | [OR #10-#26] | 13107 |
| #28 | #5 AND #9 | 86 |
| #29 | (#1 OR #2) AND #27 | 2 |
| #30 | #28 OR #29 | 87 |

**CINAHL**

9^th^ July 2020

217 records

| **#** | **Searches** | **Results** |
| --- | --- | --- |
| S1 | (MH "Muscular Dystrophy+") | 4,018 |
| S2 | duchenne* | 2,324 |
| S3 | (MH "Caregivers") | 38,008 |
| S4 | TI ( (spillover* or spill over* or carer or carers or caregiver* or caring or "care giv*" or caregiving or family or families or partner* or husband or wife or wives or spouse or spouses or spousal or child or children or son* or daughter* or offspring* or dependent* or parent* or mother* or maternal or father* or paternal or sibling* or brother* or sister* or grandparent* or grandmother* or grandfather* or close person* or relative* or "next of kin" or kinship) ) OR AB ( (spillover* or spill over* or carer or carers or caregiver* or caring or "care giv*" or caregiving or family or families or partner* or husband or wife or wives or spouse or spouses or spousal or child or children or son* or daughter* or offspring* or dependent* or parent* or mother* or maternal or father* or paternal or sibling* or brother* or sister* or grandparent* or grandmother* or grandfather* or close person* or relative* or "next of kin" or kinship) ) | 1,217,394 |
| S5 | (S1 or S2) and (S3 or S4) | 1,224 |
| S6 | TI ( ("HR-PRO" or "HRPRO" or "HRQL" or "HRQoL" or "QL" or "QoL" or “quality of life” or “health index*” or “health indices” or “health profile*” or “health status”) ) OR AB ( ("HR-PRO" or "HRPRO" or "HRQL" or "HRQoL" or "QL" or "QoL" or “quality of life” or “health index*” or “health indices” or “health profile*” or “health status”) ) | 153,996 |
| S7 | TI ( ((patient or self or child or parent or carer or proxy) N1 (appraisal* or appraised or report or reported or reporting or rated or rating* or based or assessed or assessment*)) ) OR AB ( ((patient or self or child or parent or carer or proxy) N1 (appraisal* or appraised or report or reported or reporting or rated or rating* or based or assessed or assessment*)) ) | 206,185 |
| S8 | TI ( ((disability or function or functional or functions or subjective or utility or utilities or wellbeing or well being) N2 (index or indices or instrument or instruments or measure or measures or questionnaire* or profile or profiles or scale or scales or score or scores or status or survey or surveys)) ) OR AB ( ((disability or function or functional or functions or subjective or utility or utilities or wellbeing or well being) N2 (index or indices or instrument or instruments or measure or measures or questionnaire* or profile or profiles or scale or scales or score or scores or status or survey or surveys)) ) | 69,134 |
| S9 | S6 or S7 or S8 | 383,109 |
| S10 | TI ( ("world health organization quality of life" or whoqol) ) OR AB ( ("world health organization quality of life" or whoqol) ) | 2,063 |
| S11 | TI ( (“euro qual” or “euro qual5d” or “euro qol5d” or “eq-5d” or “eq5-d” or “eq5d” or euroqual or euroqol or “euroqual5d” or “euroqol5d”) ) OR AB ( (“euro qual” or “euro qual5d” or “euro qol5d” or “eq-5d” or “eq5-d” or “eq5d” or euroqual or euroqol or “euroqual5d” or “euroqol5d”) ) | 4,478 |
| S12 | TI ( (“zarit caregiver burden interview” or “zarit burden interview” or zbi) ) OR AB ( (“zarit caregiver burden interview” or “zarit burden interview” or zbi) ) | 472 |
| S13 | TI ( (“pittsburgh sleep quality index” or psqi) ) OR AB ( (“pittsburgh sleep quality index” or psqi) ) | 2,427 |
| S14 | TI ( (“child health questionnaire parent form” or “child health questionnaire parent-form” or “chq-pf50”) ) OR AB ( (“child health questionnaire parent form” or “child health questionnaire parent-form” or “chq-pf50”) ) | 59 |
| S15 | TI ( (“sf12” or “sf 12” or “sf twelve” or sftwelve) ) OR AB ( (“sf12” or “sf 12” or “sf twelve” or sftwelve) ) | 314 |
| S16 | TI ( (“family problems questionnaire” or fpq) ) OR AB ( (“family problems questionnaire” or fpq) ) | 39 |
| S17 | TI ( (“muscular dystrophy care schedule” or md-cs or mdcs) ) OR AB ( (“muscular dystrophy care schedule” or md-cs or mdcs) ) | 861 |
| S18 | TI ( (“female sexual function index” or fsfi) ) OR AB ( (“female sexual function index” or fsfi) ) | 702 |
| S19 | TI ( (“caregiver strain index” or csi) ) OR AB ( (“caregiver strain index” or csi) ) | 981 |
| S20 | TI ( (“self-rated burden” or srb) ) OR AB ( (“self-rated burden” or srb) ) | 297 |
| S21 | TI ( (“care-related quality of life instrument” or carerqol) ) OR AB ( (“care-related quality of life instrument” or carerqol) ) | 30 |
| S22 | TI ( (“family strain questionnaire” or fsq) ) OR AB ( (“family strain questionnaire” or fsq) ) | 60 |
| S23 | TI ( (“family burden assessment scale” or fbas) ) OR AB ( (“family burden assessment scale” or fbas) ) | 138 |
| S24 | TI ( (“cope inventory” or “coping with problems experienced” or “coping orientation to problems experienced” or “brief-cope” or “brief cope”) ) OR AB ( (“cope inventory” or “coping with problems experienced” or “coping orientation to problems experienced” or “brief-cope” or “brief cope”) ) | 425 |
| S25 | TI ( (“caregiver well-being scale” or cwbs) ) OR AB ( (“caregiver well-being scale” or cwbs) ) | 71 |
| S26 | TI ( (“state-trait anxiety inventory” or stai) ) OR AB ( (“state-trait anxiety inventory” or stai) ) | 2,801 |
| S27 | S10 OR S11 OR S12 OR S13 OR S14 OR S15 OR S16 OR S17 OR S18 OR S19 OR S20 OR S21 OR S22 OR S23 OR S24 OR S25 OR S26 | 15,885 |
| S28 | S5 AND S9 | 209 |
| S29 | (S1 or S2) AND S27 | 14 |
| S30 | S28 OR S29 | 217 |

**APA PsycInfo 1806 to June Week 5 2020**

8^th^ July 2020

120 records

| **#** | **Searches** | **Results** |
| --- | --- | --- |
| 1 | Muscular Dystrophy/ | 1381 |
| 2 | duchenne*.mp. | 851 |
| 3 | exp Caregivers/ or (spillover* or spill over* or carer or carers or caregiver* or caring or “care giv*” or caregiving or family or families or partner* or husband or wife or wives or spouse or spouses or spousal or child or children or son* or daughter* or offspring* or dependent* or parent* or mother* or maternal or father* or paternal or sibling* or brother* or sister* or grandparent* or grandmother* or grandfather* or close person* or relative* or “next of kin” or kinship).tw. | 1479957 |
| 4 | (1 or 2) and 3 | 692 |
| 5 | (HR-PRO or HRPRO or HRQL or HRQoL or QL or QoL).ti,ab. or quality of life.mp. or (health index* or health indices or health profile*).ti,ab. or health status.mp. or ((patient or self or child or parent or carer or proxy) adj (appraisal* or appraised or report or reported or reporting or rated or rating* or based or assessed or assessment*)).ti,ab. or ((disability or function or functional or functions or subjective or utility or utilities or wellbeing or well being) adj2 (index or indices or instrument or instruments or measure or measures or questionnaire* or profile or profiles or scale or scales or score or scores or status or survey or surveys)).ti,ab. | 278439 |
| 6 | (world health organization quality of life or whoqol).mp. | 3571 |
| 7 | (euro qual or euro qual5d or euro qol5d or eq-5d or eq5-d or eq5d or euroqual or euroqol or euroqual5d or euroqol5d).mp. | 3324 |
| 8 | (zarit caregiver burden interview or zarit burden interview or zbi).mp. | 956 |
| 9 | (pittsburgh sleep quality index or psqi).mp. | 5441 |
| 10 | (child health questionnaire parent form or child health questionnaire parent-form or chq-pf50).mp. | 108 |
| 11 | (sf12 or sf 12 or sf twelve or sftwelve).mp. | 2011 |
| 12 | (family problems questionnaire or fpq).mp. | 66 |
| 13 | (muscular dystrophy care schedule or md-cs or mdcs).mp. | 43 |
| 14 | (female sexual function index or fsfi).mp. | 1095 |
| 15 | (caregiver strain index or csi).mp. | 1026 |
| 16 | (self-rated burden or srb).mp. | 169 |
| 17 | (care-related quality of life instrument or carerqol).mp. | 25 |
| 18 | (family strain questionnaire or fsq).mp. | 97 |
| 19 | (family burden assessment scale or fbas).mp. | 84 |
| 20 | (cope inventory or coping with problems experienced or coping orientation to problems experienced or brief-cope or brief cope).mp. | 2233 |
| 21 | (caregiver well-being scale or cwbs).mp. | 139 |
| 22 | (state-trait anxiety inventory or stai).mp. | 20500 |
| 23 | or/6-22 | 39510 |
| 24 | 4 and 5 | 105 |
| 25 | (1 or 2) and 23 | 22 |
| 26 | 24 or 25 | 120 |

1. **STAGE 2 SEARCH (November 2020)**

| **Host** | **Database** | **Dates covered** | **Results** | **Results without COSMIN filter*** |
| --- | --- | --- | --- | --- |
| Ovid | MEDLINE | 1946 to Present | 26 | 48 |
| Ovid | Embase | 1974 to Present | 42 | 102 |
| Wiley | Cochrane Database of Systematic Reviews | CDSR 1996-Present | 2 | NA |
| Wiley | Cochrane Central Register of Controlled Trials | CENTRAL 1898-Present | 4 | NA |
| EBSCO | CINAHL | 1974 to Present | 5 | 22 |
| Ovid | PsycINFO | 1806 to Present | 2 | 24 |
|  | Total retrieved | - | 81 | 196 |
|  | **Unique records in database** | **-** | **57** | NA |

*COSMIN search filter is unavailable for Cochrane Library.

**COSMIN filter source:**

Terwee CB, Jansma EP, Riphagen II, de Vet HC. Development of a methodological PubMed search filter for finding studies on measurement properties of measurement instruments. Qual Life Res. 2009 Oct;18(8):1115-23. doi: 10.1007/s11136-009-9528-5. https://www.cosmin.nl/tools/pubmed-search-filters/ [Accessed November 2020]

**Ovid MEDLINE(R) and Epub Ahead of Print, In-Process & Other Non-Indexed Citations, Daily and Versions(R) 1946 to October 30, 2020**

2^nd^ November 2020

26 records

| **#** | **Searches** | **Results** |
| --- | --- | --- |
| 1 | Muscular Dystrophy/ | 13774 |
| 2 | duchenne*.mp. | 12996 |
| 3 | exp caregivers/ or (spillover* or spill over* or carer or carers or caregiver* or caring or “care giv*” or caregiving or family or families or partner* or husband or wife or wives or spouse or spouses or spousal or child or children or son* or daughter* or offspring* or dependent* or parent* or mother* or maternal or father* or paternal or sibling* or brother* or sister* or grandparent* or grandmother* or grandfather* or close person* or relative* or “next of kin” or kinship).tw. | 5454403 |
| 4 | (1 or 2) and 3 | 5548 |
| 5 | (sf12 or sf 12 or sf-12 or short form 12 or shortform 12 or sf twelve or sftwelve or shortform twelve or short form twelve).mp. | 6063 |
| 6 | (sf36 or sf 36 or sf-36 or short form 36 or shortform 36 or sf thirtysix or sf thirty six or shortform thirtysix or shortform thirty six or short form thirtysix or short form thirty six).mp. | 26261 |
| 7 | (“beck depression inventory” or “beck inventory” or bdi).mp. | 15025 |
| 8 | (“caregiver strain index” or csi).mp. | 3725 |
| 9 | (“caregiver well-being scale” or cwbs).mp. | 31 |
| 10 | (“care-related quality of life instrument” or carerqol*).mp. | 49 |
| 11 | (“duke health proﬁle*” or dhp* or duke).mp. | 13195 |
| 12 | (“enrichd social support instrument” or essi).mp. | 149 |
| 13 | (“epworth sleepiness scale” or ess).mp. | 8650 |
| 14 | (euroqol or euro qol or eq5d* or eq 5d* or eq-5d*).mp. | 11693 |
| 15 | (“family apgar” or fapgar).mp. | 244 |
| 16 | (“family problems questionnaire” or fpq).mp. | 101 |
| 17 | (“family strain questionnaire” or fsq).mp. | 151 |
| 18 | (“female sexual function index” or fsfi).mp. | 2016 |
| 19 | (“hospital anxiety and depression scale” or hads).mp. | 10105 |
| 20 | (“kessler psychological distress scale” or k6).mp. | 1653 |
| 21 | (“pediatric quality of life inventory family impact module” or pedsql fim).mp. | 12 |
| 22 | (“perceived personal control questionnaire” or ppc).mp. | 3685 |
| 23 | (“pittsburg sleep quality index” or psqi).mp. | 3460 |
| 24 | (“psychological adaptation scale” or pas).mp. | 30240 |
| 25 | (“questionnaire on resources and stress” or qrs).mp. | 17522 |
| 26 | (“satisfaction with life scale” or swls).mp. | 1059 |
| 27 | (“social networks questionnaire” or snq).mp. | 84 |
| 28 | (“state-trait anxiety inventory form x” or stai-x).mp. | 31 |
| 29 | (“symptom checklist 90-revised” or scl-90-r).mp. | 1950 |
| 30 | (“world health organization quality of life-bref” or whoqol-bref).mp. | 2454 |
| 31 | “worry about care for child”.mp. | 0 |
| 32 | (“zarit burden interview” or zbi).mp. | 686 |
| 33 | or/5-32 | 151466 |
| 34 | 4 and 33 | 48 |
| 35 | (instrumentation or methods).fs. | 4187919 |
| 36 | (Validation Studies or Comparative Study).pt. | 1874180 |
| 37 | exp Psychometrics/ | 76307 |
| 38 | psychometr*.ti,ab. | 47668 |
| 39 | (clinimetr* or clinometr*).tw. | 1148 |
| 40 | outcome assessment.ti,ab. | 3989 |
| 41 | outcome measure*.tw. | 231604 |
| 42 | exp Observer Variation/ | 42764 |
| 43 | observer variation.ti,ab. | 1088 |
| 44 | exp Health Status Indicators/ | 309899 |
| 45 | reproducibility of results/ | 403299 |
| 46 | reproducib*.ti,ab. | 163496 |
| 47 | exp Discriminant Analysis/ | 10752 |
| 48 | (reliab* or unreliab* or valid* or coefficient or homogeneity or homogeneous or internal consistency).ti,ab. | 1445189 |
| 49 | (cronbach* and (alpha or alphas)).ti,ab. | 23696 |
| 50 | (item and (correlation* or selection* or reduction*)).ti,ab. | 23090 |
| 51 | (agreement or precision or imprecision or precise values or test-retest).ti,ab. | 432277 |
| 52 | (test and retest).ti,ab. | 28071 |
| 53 | (reliab* and (test or retest)).ti,ab. | 92329 |
| 54 | (stability or interrater or inter-rater or intrarater or intra-rater or intertester or inter-tester or intratester or intra-tester or interobserver or inter-observer or intraobserver or intraobserver or intertechnician or inter-technician or intratechnician or intra-technician).ti,ab. | 481922 |
| 55 | (interexaminer or inter-examiner or intraexaminer or intra-examiner or interassay or interassay or intraassay or intra-assay).ti,ab. | 8534 |
| 56 | (interindividual or inter-individual or intraindividual or intra-individual or interparticipant or intera-participant or intraparticipant or intra-participant or kappa* or repeatab*).ti,ab. | 253593 |
| 57 | (generaliza* or generalisa* or concordance).ti,ab. | 92348 |
| 58 | (intraclass and correlation*).ti,ab. | 26141 |
| 59 | (discriminative or known group or factor analysis or factor analyses or dimension* or subscale*).ti,ab. | 678347 |
| 60 | (multitrait and scaling and (analysis or analyses)).ti,ab. | 141 |
| 61 | (item discriminant or interscale correlation* or error or errors or individual variability).ti,ab. | 317998 |
| 62 | (variability and (analysis or values)).ti,ab. | 101823 |
| 63 | (uncertainty and (measurement or measuring)).ti,ab. | 8130 |
| 64 | (standard error of measurement or sensitiv* or responsive*).ti,ab. | 1612934 |
| 65 | ((minimal or minimally or clinical or clinically) and (important or significant or detectable) and (change or difference)).ti,ab. | 242863 |
| 66 | (small* and (real or detectable) and (change or difference)).ti,ab. | 7690 |
| 67 | (meaningful change or ceiling effect or floor effect or Item response model or irt or rasch or differential item functioning or dif or computer adaptive testing or item bank or cross-cultural equivalence).ti,ab. | 13816 |
| 68 | or/35-67 | 9298463 |
| 69 | 34 and 68 | 26 |

**Embase 1974 to 2020 October 29**

2^nd^ November 2020

42 records

| **#** | **Searches** | **Results** |
| --- | --- | --- |
| 1 | muscular dystrophy/ | 15682 |
| 2 | duchenne*.mp. | 20570 |
| 3 | exp caregiver/ or (spillover* or spill over* or carer or carers or caregiver* or caring or “care giv*” or caregiving or family or families or partner* or husband or wife or wives or spouse or spouses or spousal or child or children or son* or daughter* or offspring* or dependent* or parent* or mother* or maternal or father* or paternal or sibling* or brother* or sister* or grandparent* or grandmother* or grandfather* or close person* or relative* or “next of kin” or kinship).tw. | 6784775 |
| 4 | (1 or 2) and 3 | 9182 |
| 5 | (sf12 or sf 12 or sf-12 or short form 12 or shortform 12 or sf twelve or sftwelve or shortform twelve or short form twelve).mp. | 11512 |
| 6 | (sf36 or sf 36 or sf-36 or short form 36 or shortform 36 or sf thirtysix or sf thirty six or shortform thirtysix or shortform thirty six or short form thirtysix or short form thirty six).mp. | 49674 |
| 7 | (“beck depression inventory” or “beck inventory” or bdi).mp. | 32258 |
| 8 | (“caregiver strain index” or csi).mp. | 6191 |
| 9 | (“caregiver well-being scale” or cwbs).mp. | 32 |
| 10 | (“care-related quality of life instrument” or carerqol*).mp. | 64 |
| 11 | (“duke health proﬁle*” or dhp* or duke).mp. | 18803 |
| 12 | (“enrichd social support instrument” or essi).mp. | 124 |
| 13 | (“epworth sleepiness scale” or ess).mp. | 18753 |
| 14 | (euroqol or euro qol or eq5d* or eq 5d* or eq-5d*).mp. | 22133 |
| 15 | (“family apgar” or fapgar).mp. | 309 |
| 16 | (“family problems questionnaire” or fpq).mp. | 139 |
| 17 | (“family strain questionnaire” or fsq).mp. | 224 |
| 18 | (“female sexual function index” or fsfi).mp. | 5068 |
| 19 | (“hospital anxiety and depression scale” or hads).mp. | 22380 |
| 20 | (“kessler psychological distress scale” or k6).mp. | 2092 |
| 21 | (“pediatric quality of life inventory family impact module” or pedsql fim).mp. | 24 |
| 22 | (“perceived personal control questionnaire” or ppc).mp. | 5254 |
| 23 | (“pittsburg sleep quality index” or psqi).mp. | 7561 |
| 24 | (“psychological adaptation scale” or pas).mp. | 44078 |
| 25 | (“questionnaire on resources and stress” or qrs).mp. | 38942 |
| 26 | (“satisfaction with life scale” or swls).mp. | 1749 |
| 27 | (“social networks questionnaire” or snq).mp. | 113 |
| 28 | (“state-trait anxiety inventory form x” or stai-x).mp. | 52 |
| 29 | (“symptom checklist 90-revised” or scl-90-r).mp. | 2761 |
| 30 | (“world health organization quality of life-bref” or whoqol-bref).mp. | 3703 |
| 31 | “worry about care for child”.mp. | 0 |
| 32 | (“zarit burden interview” or zbi).mp. | 1173 |
| 33 | or/5-32 | 274808 |
| 34 | 4 and 33 | 102 |
| 35 | exp intermethod comparison/ | 267106 |
| 36 | exp data collection method/ | 1110741 |
| 37 | exp validation study/ | 86512 |
| 38 | exp feasibility study/ | 138631 |
| 39 | exp pilot study/ | 159776 |
| 40 | exp psychometry/ | 93335 |
| 41 | exp reproducibility/ | 221666 |
| 42 | exp observer variation/ | 20191 |
| 43 | exp observer variation/ | 20191 |
| 44 | exp discriminant analysis/ | 20321 |
| 45 | exp validity/ | 103350 |
| 46 | (reproducib* or audit or psychometr* or clinimetr* or clinometr* or reliab* or valid* or coefficient or internal consistency).ti,ab. | 1996596 |
| 47 | (cronbach* and (alpha or alphas)).ti,ab. | 29157 |
| 48 | (item correlation or item correlations or item selection or item selections or item reduction or item reductions or agreement or precision or imprecision or precise values or test-retest).tw. | 525885 |
| 49 | (test and retest).tw. | 34449 |
| 50 | (reliab* and (test or retest)).tw. | 124419 |
| 51 | (intraclass and correlation*).tw. | 31941 |
| 52 | (stability or interrater or inter-rater or intrarater or intra-rater or intertester or inter-tester or intratester or intratester or interobeserver or inter-observer or intraobserver or intraobserver or intertechnician or inter-technician or intratechnician or intratechnician or interexaminer or inter-examiner or intraexaminer or intraexaminer or interassay or inter-assay or intraassay or intra-assay or interindividual or inter-individual or intraindividual or intra-individualor interparticipant or inter-participant or intraparticipant or intraparticipant or kappa or kappas or coefficient of variation or repeatab*).tw. | 758224 |
| 53 | ((replicab* or repeated) and (measure or measures or findings or result or results or test or tests)).tw. | 296517 |
| 54 | (generaliza* or generalisa* or concordance or discriminative or known group or factor analysis or factor analyses or factor structure or factor structures or dimensionality or subscale* or multitrait scaling analysis or multitrait scaling analyses or item discriminant or interscale correlation or interscale correlations).tw. | 276694 |
| 55 | ((error or errors) and (measure* or correlat* or evaluat* or accuracy or accurate orprecision or mean)).tw. | 238085 |
| 56 | (individual variability or interval variability or rate variability or variability analysis).tw. | 40593 |
| 57 | (uncertainty and (measurement or measuring)).tw. | 9470 |
| 58 | (standard error of measurement or sensitiv* or responsive* or minimal detectable concentration or interpretab*).tw. | 2034133 |
| 59 | (limit and detection).tw. | 112205 |
| 60 | (small* and (real or detectable) and (change or difference)).tw. | 12292 |
| 61 | (meaningful change or minimal important change or minimal important difference or minimally important change or minimally important difference or minimal detectable change or minimal detectable difference or minimally detectable change or minimally detectable difference or minimal real change or minimal real difference or minimally real change or minimally real difference or ceiling effect or floor effect or item response model or irt or rasch or differential item functioning or dif or computer adaptive testing or item bank or cross-cultural equivalence).tw. | 22593 |
| 62 | or/35-61 | 6417712 |
| 63 | 34 and 62 | 42 |

**Cochrane Database of Systematic Reviews**

**Cochrane Central Register of Controlled Trials**

2^nd^ November 2020

6 records

| **#** | **Searches** | **Results** |
| --- | --- | --- |
| #1 | MeSH descriptor: [Muscular Dystrophies] explode all trees | 455 |
| #2 | duchenne*:ti,ab,kw | 741 |
| #3 | MeSH descriptor: [Caregivers] explode all trees | 2105 |
| #4 | (spillover* or spill over* or carer or carers or caregiver* or caring or “care giv*” or caregiving or family or families or partner* or husband or wife or wives or spouse or spouses or spousal or child or children or son* or daughter* or offspring* or dependent* or parent* or mother* or maternal or father* or paternal or sibling* or brother* or sister* or grandparent* or grandmother* or grandfather* or close person* or relative* or “next of kin” or kinship):ti,ab,kw | 354244 |
| #5 | (#1 or #2) and (#3 or #4) | 452 |
| #6 | (sf12 or “sf 12” or sf-12 or “short form 12” or “shortform 12” or “sf twelve” or “sftwelve” or “shortform twelve” or “short form twelve”):ti,ab,kw | 2712 |
| #7 | (sf36 or “sf 36” or sf-36 or “short form 36” or “shortform 36” or “sf thirtysix” or “sf thirty six” or “shortform thirtysix” or “shortform thirty six” or “short form thirtysix” or “short form thirty six”):ti,ab,kw | 13311 |
| #8 | (“beck depression inventory” or “beck inventory” or bdi):ti,ab,kw | 5803 |
| #9 | (“caregiver strain index” or csi):ti,ab,kw | 530 |
| #10 | (“caregiver well-being scale” or cwbs):ti,ab,kw | 1 |
| #11 | (“care-related quality of life instrument” or carerqol*):ti,ab,kw | 16 |
| #12 | (“duke health proﬁle*” or dhp* or duke):ti,ab,kw | 860 |
| #13 | (“enrichd social support instrument” or essi):ti,ab,kw | 23 |
| #14 | (“epworth sleepiness scale” or ess):ti,ab,kw | 2335 |
| #15 | (euroqol or euro qol or eq5d* or eq 5d* or eq-5d*):ti,ab,kw | 9202 |
| #16 | (“family apgar” or fapgar):ti,ab,kw | 25 |
| #17 | (“family problems questionnaire” or fpq):ti,ab,kw | 13 |
| #18 | (“family strain questionnaire” or fsq):ti,ab,kw | 40 |
| #19 | (“female sexual function index” or fsfi):ti,ab,kw | 905 |
| #20 | (“hospital anxiety and depression scale” or hads):ti,ab,kw | 4373 |
| #21 | (“kessler psychological distress scale” or k6):ti,ab,kw | 179 |
| #22 | (“pediatric quality of life inventory family impact module” or pedsql fim):ti,ab,kw | 6 |
| #23 | (“perceived personal control questionnaire” or ppc):ti,ab,kw | 401 |
| #24 | (“pittsburg sleep quality index” or psqi):ti,ab,kw | 1949 |
| #25 | (“psychological adaptation scale” or pas):ti,ab,kw | 1304 |
| #26 | (“questionnaire on resources and stress” or qrs):ti,ab,kw | 1768 |
| #27 | (“satisfaction with life scale” or swls):ti,ab,kw | 226 |
| #28 | (“social networks questionnaire” or snq):ti,ab,kw | 8 |
| #29 | (“state-trait anxiety inventory form x” or stai-x):ti,ab,kw | 8 |
| #30 | (“symptom checklist 90-revised” or “scl-90-r”):ti,ab,kw | 357 |
| #31 | (“world health organization quality of life-bref” or whoqol-bref):ti,ab,kw | 694 |
| #32 | worry about care for child | 0 |
| #33 | (“zarit burden interview” or zbi):ti,ab,kw | 185 |
| #34 | [OR #6-#33] | 41795 |
| #35 | #5 and #34 | 6 |

**CINAHL**

2^nd^ November 2020

5 records

| **#** | **Searches** | **Results** |
| --- | --- | --- |
| S1 | (MH “Muscular Dystrophy+”) | 3,919 |
| S2 | duchenne* | 2,237 |
| S3 | (MH “Caregivers”) | 35,683 |
| S4 | TI ( (spillover* or spill over* or carer or carers or caregiver* or caring or “care giv*” or caregiving or family or families or partner* or husband or wife or wives or spouse or spouses or spousal or child or children or son* or daughter* or offspring* or dependent* or parent* or mother* or maternal or father* or paternal or sibling* or brother* or sister* or grandparent* or grandmother* or grandfather* or close person* or relative* or “next of kin” or kinship) ) OR AB ( (spillover* or spill over* or carer or carers or caregiver* or caring or “care giv*” or caregiving or family or families or partner* or husband or wife or wives or spouse or spouses or spousal or child or children or son* or daughter* or offspring* or dependent* or parent* or mother* or maternal or father* or paternal or sibling* or brother* or sister* or grandparent* or grandmother* or grandfather* or close person* or relative* or “next of kin” or kinship) ) | 1,145,543 |
| S5 | (S1 or S2) and (S3 or S4) | 1,179 |
| S6 | TI ( (sf12 or “sf 12” or sf-12 or “short form 12” or “shortform 12” or “sf twelve” or “sftwelve” or “shortform twelve” or “short form twelve”) ) OR AB ( (sf12 or sf 12 or sf-12 or “short form 12” or “shortform 12” or “sf twelve” or “sftwelve” or “shortform twelve” or “short form twelve”) ) | 2,677 |
| S7 | TI ( (sf36 or “sf 36” or sf-36 or “short form 36” or “shortform 36” or “sf thirtysix” or “sf thirty six” or “shortform thirtysix” or “shortform thirty six” or “short form thirtysix” or “short form thirty six”) ) OR AB ( (sf36 or “sf 36” or sf-36 or “short form 36” or “shortform 36” or “sf thirtysix” or “sf thirty six” or “shortform thirtysix” or “shortform thirty six” or “short form thirtysix” or “short form thirty six”) ) | 9,945 |
| S8 | TI ( (“beck depression inventory” or “beck inventory” or bdi) ) OR AB ( (“beck depression inventory” or “beck inventory” or bdi) ) | 5,417 |
| S9 | TI ( (“caregiver strain index” or csi) ) OR AB ( (“caregiver strain index” or csi) ) | 932 |
| S10 | TI ( (“caregiver well-being scale” or cwbs) ) OR AB ( (“caregiver well-being scale” or cwbs) ) | 61 |
| S11 | TI ( (“care-related quality of life instrument” or carerqol*) ) OR AB ( (“care-related quality of life instrument” or carerqol*) ) | 31 |
| S12 | TI ( (“duke health proﬁle*” or dhp* or duke) ) OR AB ( (“duke health proﬁle*” or dhp* or duke) ) | 2,489 |
| S13 | TI ( (“enrichd social support instrument” or essi) ) OR AB ( (“enrichd social support instrument” or essi) ) | 84 |
| S14 | TI ( (“epworth sleepiness scale” or ess) ) OR AB ( (“epworth sleepiness scale” or ess) ) | 3,057 |
| S15 | TI ( (euroqol or euro qol or eq5d* or eq 5d* or eq-5d*) ) OR AB ( (euroqol or euro qol or eq5d* or eq 5d* or eq-5d*) ) | 5,158 |
| S16 | TI ( (“family apgar” or fapgar) ) OR AB ( (“family apgar” or fapgar) ) | 148 |
| S17 | TI ( (“family problems questionnaire” or fpq) ) OR AB ( (“family problems questionnaire” or fpq) ) | 41 |
| S18 | TI ( (“family strain questionnaire” or fsq) ) OR AB ( (“family strain questionnaire” or fsq) ) | 56 |
| S19 | TI ( (“female sexual function index” or fsfi) ) OR AB ( (“female sexual function index” or fsfi) ) | 687 |
| S20 | TI ( (“hospital anxiety and depression scale”) ) OR AB ( (“hospital anxiety and depression scale”) ) | 3,916 |
| S21 | TI ( (“kessler psychological distress scale” or k6) ) OR AB ( (“kessler psychological distress scale” or k6) ) | 374 |
| S22 | TI ( (“pediatric quality of life inventory family impact module” or pedsql fim) ) OR AB ( (“pediatric quality of life inventory family impact module” or pedsql fim) ) | 14 |
| S23 | TI ( (“perceived personal control questionnaire” or ppc) ) OR AB ( (“perceived personal control questionnaire” or ppc) ) | 773 |
| S24 | TI ( (“pittsburg sleep quality index” or psqi) ) OR AB ( (“pittsburg sleep quality index” or psqi) ) | 1,496 |
| S25 | TI ( (“psychological adaptation scale” or pas) ) OR AB ( (“psychological adaptation scale” or pas) ) | 8,003 |
| S26 | TI ( (“questionnaire on resources and stress” or qrs) ) OR AB ( (“questionnaire on resources and stress” or qrs) ) | 4,992 |
| S27 | TI ( (“satisfaction with life scale” or swls) ) OR AB ( (“satisfaction with life scale” or swls) ) | 766 |
| S28 | TI ( (“social networks questionnaire” or snq) ) OR AB ( (“social networks questionnaire” or snq) ) | 45 |
| S29 | TI ( (“state-trait anxiety inventory form x” or stai-x) ) OR AB ( (“state-trait anxiety inventory form x” or stai-x) ) | 6 |
| S30 | TI ( (“symptom checklist 90-revised” or scl-90-r) ) OR AB ( (“symptom checklist 90-revised” or scl-90-r) ) | 482 |
| S31 | TI ( (“world health organization quality of life-bref” or whoqol-bref) ) OR AB ( (“world health organization quality of life-bref” or whoqol-bref) ) | 1,325 |
| S32 | TI “worry about care for child” OR AB “worry about care for child” | 529 |
| S33 | TI ( (“zarit burden interview” or zbi) ) OR AB ( (“zarit burden interview” or zbi) ) | 415 |
| S34 | S6 or S7 or S8 or S9 or S10 OR S11 OR S12 OR S13 OR S14 OR S15 OR S16 OR S17 OR S18 OR S19 OR S20 OR S21 OR S22 OR S23 OR S24 OR S25 OR S26 or S27 or S28 or S29 or S30 or S31 or S32 or S33 | 50,269 |
| S35 | S5 and S34 | 22 |
| S36 | (MH “Psychometrics”) or ( TI psychometr* or AB psychometr* ) or ( TI clinimetr* or AB clinimetr* ) or ( TI clinometr* OR AB clinometr* ) or (MH “Outcome Assessment”) or ( TI outcome assessment or AB outcome assessment ) or ( TI outcome measure* or AB outcome measure* ) or (MH “Health Status Indicators”) or (MH “Reproducibility of Results”) or (MH “Discriminant Analysis”) or ( ( TI reproducib* or AB reproducib* ) or ( TI reliab* or AB reliab* ) or ( TI unreliab* or AB unreliab* ) ) or ( ( TI valid* or AB valid* ) or ( TI coefficient or AB coefficient ) or ( TI homogeneity or AB homogeneity ) ) or ( TI homogeneous or AB homogeneous ) or ( TI “coefficient of variation” or AB “coefficient of variation” ) or ( TI “internal consistency” or AB “internal consistency” ) or (MH “Internal Consistency+”) or (MH “Reliability+”) or (MH “Measurement Error+”) or (MH “Content Validity+”) or “hypothesis testing” or “structural validity” or “cross-cultural validity” or (MH “Criterion-Related Validity+”) or “responsiveness” or “interpretability” or ( TI reliab* or AB reliab* ) and ( (TI test or AB test) OR (TI retest or AB retest) ) or ( TI stability or AB stability ) or ( TI interrater or AB interrater ) or ( TI inter-rater or AB inter-rater ) or ( TI intrarater or AB intrarater ) or ( TI intra-rater or AB intrarater ) or ( TI intertester or AB intertester) or (TI inter-tester or AB inter-tester) or ( TI intratester or AB intratester) or ( TI intra-tester or AB intra-tester) or ( TI interobserver or AB interobserver) or (TI inter-observer or AB inter-observer ) or ( TI intraobserver or AB intraobserver) or ( TI intra-observer or AB intra-observer) or ( TI intertechnician or AB intertechnician) or (TI inter-technician or AB inter-technician) or ( TI intratechnician or AB intratechnician ) or ( TI intra-technician or AB intra-technician ) or ( TI interexaminer or AB interexaminer ) or (TI inter-examiner or AB inter-examiner) or (TI intraexaminer or AB intraexaminer ) OR (TI intra-examiner or AB intra-examiner ) or (TI intra-examiner or AB intraexaminer ) or (TI interassay or AB interassay ) or ( TI inter-assay or AB inter-assay ) or ( TI intraassay or AB intraassay) or ( TI intra-assay or AB intra-assay ) or (TI interindividual or AB interindividual) or (TI inter-individual or AB inter-individual) OR (TI intraindividual or AB intraindividual) or (TI intra-individual or AB intra-individual) or (TI interparticipant or AB interparticipant) or (TI inter-participant or AB inter-participant ) or (TI intraparticipant or AB intraparticipant) or (TI intra-participant or AB intra-participant ) or (TI kappa or AB kappa) or (TI kappa’s or AB kappa’s ) or (TI kappas or AB kappas) or (TI repeatab* or AB repeatab*) or ( TI responsive* or AB responsive* ) or ( TI interpretab* or AB interpretab* ) | 626,364 |
| S37 | (S35 AND S36) | 5 |

**APA PsycInfo 1806 to October Week 4 2020**

2^nd^ November 2020

2 records

| **#** | **Searches** | **Results** |
| --- | --- | --- |
| 1 | Muscular Dystrophy/ | 1390 |
| 2 | duchenne*.mp. | 862 |
| 3 | exp Caregivers/ or (spillover* or spill over* or carer or carers or caregiver* or caring or “care giv*” or caregiving or family or families or partner* or husband or wife or wives or spouse or spouses or spousal or child or children or son* or daughter* or offspring* or dependent* or parent* or mother* or maternal or father* or paternal or sibling* or brother* or sister* or grandparent* or grandmother* or grandfather* or close person* or relative* or “next of kin” or kinship).tw. | 1500253 |
| 4 | (1 or 2) and 3 | 698 |
| 5 | (sf12 or sf 12 or sf-12 or short form 12 or shortform 12 or sf twelve or sftwelve or shortform twelve or short form twelve).mp. | 2658 |
| 6 | (sf36 or sf 36 or sf-36 or short form 36 or shortform 36 or sf thirtysix or sf thirty six or shortform thirtysix or shortform thirty six or short form thirtysix or short form thirty six).mp. | 10025 |
| 7 | (“beck depression inventory” or “beck inventory” or bdi).mp. | 45771 |
| 8 | (“caregiver strain index” or csi).mp. | 1049 |
| 9 | (“caregiver well-being scale” or cwbs).mp. | 144 |
| 10 | (“care-related quality of life instrument” or carerqol*).mp. | 29 |
| 11 | (“duke health proﬁle*” or dhp* or duke).mp. | 2978 |
| 12 | (“enrichd social support instrument” or essi).mp. | 171 |
| 13 | (“epworth sleepiness scale” or ess).mp. | 4674 |
| 14 | (euroqol or euro qol or eq5d* or eq 5d* or eq-5d*).mp. | 3526 |
| 15 | (“family apgar” or fapgar).mp. | 228 |
| 16 | (“family problems questionnaire” or fpq).mp. | 66 |
| 17 | (“family strain questionnaire” or fsq).mp. | 97 |
| 18 | (“female sexual function index” or fsfi).mp. | 1124 |
| 19 | (“hospital anxiety and depression scale” or hads).mp. | 12066 |
| 20 | (“kessler psychological distress scale” or k6).mp. | 1726 |
| 21 | (“pediatric quality of life inventory family impact module” or pedsql fim).mp. | 6 |
| 22 | (“perceived personal control questionnaire” or ppc).mp. | 1061 |
| 23 | (“pittsburg sleep quality index” or psqi).mp. | 1421 |
| 24 | (“psychological adaptation scale” or pas).mp. | 5873 |
| 25 | (“questionnaire on resources and stress” or qrs).mp. | 387 |
| 26 | (“satisfaction with life scale” or swls).mp. | 7092 |
| 27 | (“social networks questionnaire” or snq).mp. | 26 |
| 28 | (“state-trait anxiety inventory form x” or stai-x).mp. | 38 |
| 29 | (“symptom checklist 90-revised” or scl-90-r).mp. | 7754 |
| 30 | (“world health organization quality of life-bref” or whoqol-bref).mp. | 2249 |
| 31 | “worry about care for child”.mp. | 0 |
| 32 | (“zarit burden interview” or zbi).mp. | 888 |
| 33 | or/5-32 | 102832 |
| 34 | 4 and 33 | 24 |
| 35 | (psychometr* or clinimetr* or ‎clinometr* or outcome ‎assessment or outcome ‎measure*‎ or ‎‎observer variation ‎or reproducib* or reliab*‎ or ‎unreliab* or valid* or coefficient ‎or ‎homogeneity or homogeneous ‎‎or ‎‎internal consistency or ‎agreement or precision or ‎imprecision ‎or precise ‎values ‎or test-retest or reliab* or ‎stability or interrater or inter-rater ‎‎or intrarater or ‎intra-rater or ‎intertester or inter-‎tester or ‎intratester ‎or intra-tester or ‎interobserver or inter-‎observer or ‎intraobserver ‎or intra-‎observer or ‎intertechnician or inter-technician ‎or intratechnician ‎or intra-‎technician ‎or interexaminer or ‎inter-examiner or intraexaminer ‎or intra-examiner or ‎interassay ‎or ‎inter-assay or intraassay or ‎intra-assay or ‎interindividual or ‎inter-individual ‎or ‎intraindividual ‎or intra-individual or ‎interparticipant or inter-participant ‎or intraparticipant ‎or ‎intra-‎participant or ‎kappa* or repeatab*‎ or ‎generaliza* or generalisa* or ‎concordance or discriminative or ‎‎known group or factor analys* ‎or dimension* or ‎subscale* ‎or ‎item discriminant ‎or interscale ‎correlation* or error* or ‎individual variability or standard ‎error of ‎measurement or sensitiv* ‎or responsive* or meaningful ‎change or ‎‎ceiling effect or ‎‎‎floor effect or item response ‎model or irt or rasch or ‎differential ‎item functioning or ‎dif or ‎‎computer adaptive testing‎ or item bank or cross-cultural ‎‎equivalence‎).mp. | 665817 |
| 36 | (‎cronbach* ‎alpha* or replicab* measure* ‎or replicab* finding* or ‎replicab* ‎result* or ‎‎replicab* ‎test* or repeated measure* or ‎‎repeated finding* or repeated ‎result* or ‎‎repeated test* or ‎item correlation* ‎or item ‎selection* or item reduction* ‎or test retest ‎or intraclass ‎correlation* or multitrait scaling ‎analys* or uncertainty measur* ‎or variability ‎analys* or ‎variability value* or minimal* ‎important change or minimal* ‎important difference ‎or minimal* ‎significant change or minimal* ‎significant difference or minimal* ‎detectable ‎change or minimal* ‎detectable difference or clinical* ‎important change or clinical* ‎important ‎difference or clinical* ‎significant change or clinical* ‎significant difference or clinical* ‎detectable ‎change or clinical* ‎detectable difference or small* ‎real change or small* real ‎difference or ‎‎small* detectable ‎change or small* detectable ‎difference‎).mp. | 32957 |
| 37 | 35 or 36 | 681120 |
| 38 | 34 and 37 | 2 |

Modified COSMIN PscyINFO filter for PsycINFO via Ovid (statements 35-36)
