# Supplementary material for: Measuring carer quality of life in Duchenne muscular dystrophy: a systematic review of the reliability and validity of self-report instruments using COSMIN
Source: Health Qual Life Outcomes. 2022 Apr 2;20:57. doi: 10.1186/s12955-022-01964-4 (PMC8977045; doi:10.1186/s12955-022-01964-4)
Supplement: Supplementary file 3 — Additional file 3: Instructions for carer ratings of instruments’ content validity [file 12955_2022_1964_MOESM3_ESM.docx]

Article title: Measuring Carer Quality of Life in Duchenne Muscular Dystrophy: A Systematic Review of the Reliability and Validity of Self-Report Instruments Using COSMIN

Journal name: Journal of Neurology

Author names: Jill Carlton, Philip A. Powell, Ruth Wong, Project HERCULES Carer Group

Corresponding author: Jill Carlton, School of Health and Related Research (ScHARR), University of Sheffield, Regent Court, 30 Regent Street, Sheffield, S1 4DA, United Kingdom, [j.carlton@sheffield.ac.uk](mailto:j.carlton@sheffield.ac.uk), +44 114 222 0779

**Online Resource 3 - Instructions for Carer Ratings of Instruments’ Content Validity**

**Instructions for rating the questionnaires**

Thank you for agreeing to collaborate with us on this project as family carers (hereafter ‘carers’) of someone with Duchenne. In this project as part of Project HERCULES we are exploring the suitability of questionnaires used to assess the quality of life of carers of people living with Duchenne. In particular, we are looking for people with caring experience to help us rate the questionnaires that have been used to get an understanding of how *good* these questionnaires are for measuring quality of life in Duchenne carers from the perspective of the carers’ themselves.

To do this we are following standard steps developed by experts and researchers. Your ratings will contribute to an overall assessment of the questionnaire, which will be written up in a publication. Your collaboration will be recognised through co-authorship on the publication as a collaborative group author (e.g. ‘the Project HERCULES carer group’ – name to be confirmed).

Each of you have been given some questionnaires to have a look at and rate. Ideally, we would like you to do this sometime in the next two weeks (but if you need more time, let us know). We will invite you to a collaborative meeting with the researcher(s) and two other carers to give your feedback and to discuss any reasons behind your ratings. The meeting will last no more than an hour.

Below we define some key terms of the exercise and describe how to rate the questionnaires on the rating sheets provided. Please note that there are no right or wrong answers here, we are interested in your views on the questionnaires.

1. **First, some key terms**

**Quality of life**

In this project we are interested in ‘carer quality of life’. Quality of life can mean different things to different people, but often incorporates physical, psychological (including emotional), and social aspects of life. Please take some time before you rate the questionnaires to reflect on and think about all the ways in which being a carer of some with Duchenne affects your life. This can be on your physical health, psychological (or emotional) health, and your social life and relationships. There may be other aspects of your life too that you think are personally relevant.

**Content validity**

The technical term for the way in which your ratings will be used to assess the questionnaire is an assessment of its ‘content validity’, or the degree to which the questionnaire measures what it proposes to measure. The ratings you will be making will be about three aspects of this.

*Relevance* refers to whether the questionnaire asks about things that are relevant to understanding carer quality of life in Duchenne.

*Comprehensiveness* refers to whether anything important is missing from the questionnaire to assess carer quality of life in Duchenne.

*Comprehensibility* refers to the extent to which the questionnaire can be understood (as intended).

1. **How to complete the rating sheet**

There are a number of attachments with this email. There should be a copy of each of the questionnaires we would like you to rate, and an Excel document. Each column has the name of a questionnaire in it, which corresponds with the questionnaires you have been sent. Please note we are interested in your ratings of the questionnaire, not your questionnaire responses. So you don’t have to fill them in, unless it helps you to do so to make your ratings.

To rate the questionnaires, there are 8 questions for you to consider. Each question is answered with either a **+**, **-** or **?**. A **+** is a positive answer. A **-** is a negative answer. A **?** means that you are uncertain, or not sure. For each question please use the drop down box to select your response (+/-/?).

We have had to write the questions on the Excel document just as they are written in the published manual (i.e. with all the technical terms). Some of the words or terms they use may not be familiar to you, so we have tried to give an explanation for each one below.

Some of the questions ask you to make a judgement across a group of questions (or ‘items’) and the guidance is whether you think ‘85%’ of the questions fulfil the criteria to give a positive rating. While advocated by researchers, this value is to a degree arbitrary and we certainly don’t want you to calculate exactly whether 85% of the questions are relevant or appropriate. Instead, please use this as a general guide, thinking more, for example, if three quarters or so of the questionnaire matches the criteria below, for you to give a positive rating.

1. *Are the included items relevant for the construct of interest?*

As mentioned above, the construct of interest for the purpose of this task is carer quality of life in Duchenne. An item is another word for question. When answering this question, consider whether at least 85% of the questions on the questionnaire are relevant to measuring or assessing the impact caring for someone with Duchenne has on the carer’s quality of life.

1. *Are the included items relevant for the target population of interest?*

When answering this question, consider whether at least 85% of the questions are relevant to carers of people with Duchenne.

1. *Are the included items relevant to the context of interest?*

When answering this question, consider whether at least 85% of the questions are relevant to being able to measure the impact caring for someone with Duchenne has on the carer’s quality of life.

1. *Are the response options appropriate?*

Response options are the answers that a person completing the questionnaire can give. Response options can be different from one questionnaire to another. Sometimes they are about frequency, such as Never/Sometimes/Always. Sometimes they are about severity, such as Little/Moderate/Extreme. Response options can differ in what they are asking about (such as frequency, severity, agreement), and also in the number of options there are. Some questionnaires have 3 response options, others have 4, 5, 6 or even 7. When answering this question, consider whether at least 85% of the response options are appropriate for measuring the impact caring for someone with Duchenne has on the carer’s quality of life.

1. *Is the recall period appropriate?*

Some questionnaires have a recall period. A recall period is the time frame that people should think about when answering the questions within the questionnaire. For example, some questionnaires may have instructions such as “We would like you to **think about the last week**…..”. The recall period used can be different from one questionnaire to another. When answering this question, consider whether the recall period is appropriate for measuring the impact caring for someone with Duchenne has on the carer’s quality of life

1. *Are all key concepts included?*

When answering this question, we would like you to think about all the ways in which caring for someone with Duchenne can impact upon the carer’s quality of life. Does the questionnaire include questions on all the main areas that you feel are important?

1. *Are the PRO items appropriately worded?*

PRO is another word for questionnaire. When answering this question, consider whether at least 85% of the items and response options are appropriately worded. Do the questions make sense? Can you understand them? Do the response options make sense?

1. *Do the response options match the question?*

When answering this question, consider whether at least 85% of the response options match the questions. Do the questions work with the response options? Do the response options make sense?

Any questions please get in-touch (p.a.powell@sheffield.ac.uk).

Please send your Excel spreadsheets back to us by email (p.a.powell@sheffield.ac.uk).

Thank you for taking the time to collaborate with us on this project.
