# Supplementary material for: Measuring carer quality of life in Duchenne muscular dystrophy: a systematic review of the reliability and validity of self-report instruments using COSMIN
Source: Health Qual Life Outcomes. 2022 Apr 2;20:57. doi: 10.1186/s12955-022-01964-4 (PMC8977045; doi:10.1186/s12955-022-01964-4)
Supplement: Supplementary file 4 — Additional file 4: Full list of questionnaires identified in the searches [file 12955_2022_1964_MOESM4_ESM.docx]

Article title: Measuring Carer Quality of Life in Duchenne Muscular Dystrophy: A Systematic Review of the Reliability and Validity of Self-Report Instruments Using COSMIN

Journal name: Journal of Neurology

Author names: Jill Carlton, Philip A. Powell, Ruth Wong, Project HERCULES Carer Group

Corresponding author: Jill Carlton, School of Health and Related Research (ScHARR), University of Sheffield, Regent Court, 30 Regent Street, Sheffield, S1 4DA, United Kingdom, [j.carlton@sheffield.ac.uk](mailto:j.carlton@sheffield.ac.uk), +44 114 222 0779

**Online Resource 4 – Full List of Questionnaires Identified in the Searches**

|  | **Instrument** | **Reviewed (Y/N)** | **Reasons for exclusion** |
| --- | --- | --- | --- |
| 1 | 12-Item Short Form Survey (SF-12) | Y |  |
| 2 | 36-Item Short Form Survey (SF-36) | Y |  |
| 3 | Beck Depression Inventory (BDI) | Y |  |
| 4 | Brief Resilient Coping Scale (BRCS) | N | Not QoL (personality / coping styles) |
| 5 | Caregiver Strain Index (CSI) | Y |  |
| 6 | Caregiver Strain Index Plus (CSI+) | Y |  |
| 7 | Caregiver Well-being Scale (CWBS) | Y |  |
| 8 | Care-related Quality of Life Instrument (CarerQol) | Y |  |
| 9 | Child Health Questionnaire Parent Form 50 Questions (CHQ-PF50) | N | No free review copy |
| 10 | Chronic Impact and Coping Instrument (CICI) | N | No free review copy |
| 11 | COPE Inventory (COPE) | N | Not QoL (personality / coping styles) |
| 12 | Coping Self-Efficacy Scale (CSE) | N | Not QoL (personality / coping styles) |
| 13 | DUKE Health Profile (DUKE) | Y |  |
| 14 | ENRICHD Social Support Instrument (ESSI) | Y |  |
| 15 | Epworth Sleepiness Scale (ESS) | Y |  |
| 16 | EQ-5D-3L | Y |  |
| 17 | EQ-5D-5L | Y |  |
| 18 | Family APGAR (FAPGAR) | Y |  |
| 19 | Family Burden Assessment Scale (BAS) | N | Duplicate same as FBIS |
| 20 | Family Burden Interview Schedule (FBIS) | N | Interview schedule (not self-report) |
| 21 | Family Environment Scale (FES) | N | No free review copy |
| 22 | Family Hardiness Index (FHI) | N | Not specific to caregiver |
| 23 | Family Inventory of Life Events and Changes (FILE) | N | Not QoL (life events) |
| 24 | Family Problems Questionnaire (FPQ) | Y |  |
| 25 | Family Strain Questionnaire (FSQ) | N | No validated English version |
| 26 | Female Sexual Function Index (FSFI) | Y |  |
| 27 | General Health Questionnaire-28 (GHQ-28) | N | No free review copy |
| 28 | General Self-efficacy scale (GSE) | N | Not QoL (personality / coping styles) |
| 29 | Hospital Anxiety and Depression Scale (HADS) | Y |  |
| 30 | Johnson & Johnson Stress Profile | N | Unpublished / inaccessible |
| 31 | Kessler Psychological Distress Scale (K6) | Y |  |
| 32 | Life Events Questionnaire (Checklist) (LEC) | N | Unpublished / inaccessible |
| 33 | Life Orientation Test-Revised (LOT-R) | N | Not QoL (personality / coping styles) |
| 34 | Life Satisfaction Index for Parents (LSI-P) | N | No free review copy |
| 35 | McMaster Family Assessment Device (FAD) | N | Not specific to caregiver |
| 36 | Muscular Dystrophy Care Schedule (MD-CS) | N | No validated English version |
| 37 | Parenting Stress Index-Short Form (PSI-SF) | N | No free review copy |
| 38 | PedsQL Family Impact Module (PedsQL FIM) | Y |  |
| 39 | Perceived Personal Control Questionnaire (PPC) | Y |  |
| 40 | Perceived Stress Scale (PSS) | Y |  |
| 41 | Pittsburg Sleep Quality Index (PSQI) | Y |  |
| 42 | Psychological Adaptation Scale (PAS) | Y |  |
| 43 | Psychosocial Adjustment and Role Skills Scale (PARS III) | N | Not specific to caregiver |
| 44 | Questionnaire on Resources and Stress (QRS) | Y |  |
| 45 | Resilience Scale for Adults (RSA) | N | Not QoL (personality / coping styles) |
| 46 | Satisfaction with Life Scale (SWLS) | Y |  |
| 47 | Self-rated Burden Scale (SRB) | N | Single-item measure |
| 48 | Social Networks Questionnaire (SNQ) | Y |  |
| 49 | State-Trait Anxiety Inventory (form X) (STAI-X) | Y |  |
| 50 | Strength and Difficulties Questionnaire (SDQ) | N | Not specific to caregiver |
| 51 | Symptom Checklist 90-Revised (SCL-90-R) | Y |  |
| 52 | The Coping Scale | N | Unpublished / inaccessible |
| 53 | Understanding of and Attitude toward Muscular Dystrophy | N | Unpublished / inaccessible |
| 54 | Ways of Coping Questionnaire (WCQ) | N | Not QoL (personality / coping styles) |
| 55 | WHO Quality of Life-BREF (WHOQOL-BREF) | Y |  |
| 56 | Work Productivity and Activity Impairment Questionnaire (WPAI) | N | Not QoL |
| 57 | Worry about Care for Child with DBMD (WAC-DBMD) | Y |  |
| 58 | Zarit Burden Inventory 22-item (ZBI 22-item) | Y |  |
| 59 | Zarit Burden Inventory 12-item (ZBI 12-item) | Y |  |
| 60 | Zarit Burden Inventory 4-item (ZBI 4-item) | Y |  |
